# Genome-wide analysis, molecular cloning and expression profiling reveal tissue-specifically expressed, feedback-regulated, stress-responsive and alternatively spliced novel genes involved in gibberellin metabolism in *Salvia miltiorrhiza*

**DOI:** 10.1186/s12864-015-2315-5

**Published:** 2015-12-21

**Authors:** Qing Du, Caili Li, Dongqiao Li, Shanfa Lu

**Affiliations:** Institute of Medicinal Plant Development, Chinese Academy of Medical Sciences & Peking Union Medical College, No.151, Malianwa North Road, Haidian District, Beijing, 100193 China

**Keywords:** Alternative splicing, Gibberellin, *Salvia miltiorrhiza*, Tanshinone, Terpenoid, Traditional Chinese Medicine

## Abstract

**Background:**

Gibberellin (GA), a classical phytohormone, plays significant roles in plant growth and development. It shares the important intermediate diphosphate precursor, GGPP, with the main lipophilic bioactive components, diterpenoid tanshinones in *Salvia miltiorrhiza* Bunge, one of the most important Traditional Chinese Medicine materials and an emerging model medicinal plant. Analysis of GA metabolism and regulation may help to demonstrate the biological functions of GAs and the crosstalk between GA metabolism and tanshinone biosynthesis in *S. miltiorrhiza*. However, genes involved in the conversion of *ent*-kaurene to GAs have not been systematically studied.

**Results:**

Through genome-wide prediction and molecular cloning, twenty two candidate gibberellin metabolism pathway genes were systematically identified for the first time. It includes a *SmKO*, two *SmKAOs*, six *SmGA20oxs*, two *SmGA3oxs* and eleven *SmGA2oxs*, of which twenty genes are novel. The deduced proteins showed sequence conservation and divergence. Gibberellin metabolism pathway genes exhibited tissue-specific expression patterns and responded differentially to exogenous GA_3_ treatment, indicating differential regulation of gibberellin metabolism in different tissue types in *S. miltiorrhiza. SmKAO1*, *SmKAO2*, *SmGA2ox2*, and *SmGA2ox4*–*SmGA2ox7* were significantly up-regulated; *SmGA20ox2, SmGA3ox1, SmGA2ox1*, *SmGA2ox8*, *SmGA2ox10* and *SmGA2ox11* were significantly down-regulated; while the responses of many other genes varied among different tissue-types and time-points of GA_3_ treatment, suggesting the complexity of feedback regulation. Tanshinone biosynthesis-related genes, such as *SmCPS1* and *SmKSL1*, were up-regulated in response to GA_3_ treatment. Among the 22 identified genes, nine responded to yeast extract and Ag^+^-treatment in *S. miltiorrhiza* hairy roots. Moreover, tissue-specifically expressed splice variants were identified for *SmKO*, *SmGA20ox3*, *SmGA2ox3* and *SmGA2ox11*, of which *SmKOv1*, *SmGA20ox3v* and *SmGA2ox11v1* were GA_3_-responsive, suggesting the importance of alternative splicing in regulating GA metabolism.

**Conclusions:**

The results show tissue-specifically expressed, feedback-regulated, stress-responsive and alternatively spliced novel genes and reveal multiple layer regulation of GA metabolism and crosstalk between gibberellin metabolism and tanshinone biosynthesis in *S. miltiorrhiza*.

**Electronic supplementary material:**

The online version of this article (doi:10.1186/s12864-015-2315-5) contains supplementary material, which is available to authorized users.

## Background

Gibberellin (GA), discovered by Dr. E. Kurosawa in 1926 [1], is a classical phytohormone. It is a large group of tetracylic diterpenoids with more than 130 members identified in plants, fungi and bacteria [2, 3]. Based on the number of carbon atoms, GAs can be classified into C_19_-GAs and C_20_-GAs, which consist of 19 and 20 carbon atoms, respectively. C_19_-GAs, such as GA_1_, GA_3_, GA_4_, GA_5_, GA_7_, GA_9_ and GA_20,_ are converted from C_20_-GAs, including GA_12_, GA_15_, GA_24_, GA_19_, GA_44_, GA_53_ and so on. GA_1_, GA_3_, GA_4_ and GA_7_ of C_19_-GAs are biologically active GAs in higher plants. GA_1_ and GA_4_ are saturated and are the main bioactive GAs with relative abundance varying in different species and tissues, while GA_3_ and GA_7_ are double bond-containing GAs with less abundance compared with GA_1_ and GA_4_ [4]. GAs play vital roles in many diverse aspects of plant growth and development, such as seed germination [5, 6], shoot elongation [7], leaf expansion [8], flower development [9], and fruit-setting [10, 11].

Similarly with other diterpenoids, GAs are synthesized from *trans*-geranylgeranyl diphosphate (GGPP), an important intermediate diphosphate precursor produced mainly from pyruvate and glyceraldehyde 3-phosphate via the 2-C-methyl-D-erythritol 4-phosphate (MEP) pathway in the plastid. The formation of GGPP during diterpenoid biosynthesis also depends on the crosstalk between the MEP pathway and the mevalonate (MVA) pathway operated in the cytoplasm, peroxisome, endoplasmic reticulum and mitochondrion [12]. GA metabolism pathway from GGPP to end-products can be generally divided into three stages [2, 13, 14]. In the first stage, *ent*-kaurene is formed from GGPP in two steps via *ent*-copalyl diphosphate (CPP) under the catalysis of *ent*-copalyl diphosphate synthase (CPS) and *ent*-kaurene synthase (KS). *ent*-Kaurene is further oxidized to GA_12_, in the second stage, by two multifunctional cytochrome P450 monooxygenases (P450s), known as *ent*-kaurene oxidase (KO) and *ent*-kaurenoic acid oxidase (KAO). KO converts *ent*-kaurene to *ent*-kaurenoic acid via *ent*-kaurenol and *ent*-kaurenal, while KAO converts *ent*-kaurenoic acid to GA_12_ via *ent*-7α-hydroxykaurenoic acid and GA_12_-aldehyde. In the last stage, bioactive GAs are produced under the catalysis of GA 20-oxidase (GA20ox) and GA 3-oxidase (GA3ox) and can be deactivation by GA 2-oxidase (GA2ox) in plants [2, 14–17].

Genes involved in GA metabolism have been identified in various plant species, such as *Arabdopsis* [16–23], pumpkin [24], rice [25], pea [6, 26, 27], and maize [28]. CPS, KS, KO and KAO enzymes involved in the early steps of the GA metabolism pathway are usually encoded by single or few genes [4]. For instance, the rice *OsCPS* and *OsKSL* gene families consist of three and eleven members, respectively; however, only *OsCPS1* and *OsKS1* are responsible for *ent*-kaurene biosynthesis. Similarly, in the *Arabidopsis* genome, only a *CPS* and a *KS* exist. KO and KAO are members of the large P450 gene family containing 246 and 356 genes in *Arabidopsis* and rice, respectively [29]. In *Arabidopsis*, there are only one *AtKO* and two *AtKAO* genes. The number of rice *OsKO* and *OsKAO* is two and one, respectively. Unlike the enzymes involved in stages one and two, GA20ox, GA3ox and GA2ox, which play catalytic roles in the third stage, are encoded by multiple differentially expressed genes [4]. *Arabidopsis* has seven *AtGA2ox*, four *AtGA3ox* and five *AtGA20ox* genes (http://www.arabidopsis.org), each of which exhibits a unique expression pattern and plays distinct developmental roles [19–21, 30, 31]. For instance, *AtGA3ox1* and *AtGA3ox2* are responsible for bioactive GA biosynthesis during vegetative growth, while *AtGA3ox1*, *AtGA3ox3* and *AtGA3ox4* are important for the development of reproductive organs [19, 21]. Among the five *AtGA20ox* genes, *AtGA20ox1*, *AtGA20ox2* and *AtGA20ox3* are the dominant paralogs [20]. *AtGA20ox3* is functionally redundant with *AtGA20ox1* and *AtGA20ox2*, while *AtGA20ox4* and *AtGA20ox5* play very minor roles in most developmental stages [20]. Differential expression and distinct developmental roles were also observed for rice 2-oxoglutarate-dependent dioxygenase (2-ODD) genes, which include eight *OsGA20ox*, two *OsGA3ox* and eleven *OsGA2ox* genes (http://www.ricedata.cn/gene) [23, 25, 31, 32]. In addition to differential expression in organs, tissues and developmental stages of plants, the expression of GA metabolism pathway genes is also regulated by environmental cues, such as light, temperature and other stresses [4, 33]. Moreover, transcript levels of some, but not all, GA metabolism pathway genes are under feedback control [18, 34–36]. It includes inhibition of some *GA20ox* and *GA3ox* gene expression and activation of some *GA2ox* gene expression [4, 15, 19].

*Salvia miltiorrhiza* Bunge is an important Traditional Chinese Medicine (TCM) material widely used in Chinese medicines. It mainly produces two groups of bioactive components, including the water-soluble phenolic acids and the lipid-soluble tanshinones. The latters are a group of diterpenoids sharing the universal precursor, GGPP, with other diterpenoids, including GAs [12, 37]. The biosynthesis of tanshinones from GGPP involves *SmCPS1*, *SmKSL1*, *CYP76AH1* and other unknown genes [12, 37, 38]. *SmCPS1* of the *S. miltiorrhiza CPS* gene family encodes enzymes responsible for the conversion of GGPP to CPP, which is subsequently cycled and rearranged to miltiradiene under the catalysis of enzymes encoded by *SmKSL1*, a member of the *SmKSL* gene family. Recently, a total of 40 genes, members of 19 gene families involved in terpenoid biosynthesis, have been identified and characterized in *S. miltiorrhiza* through a genome-wide analysis [12]. Of the 40 genes, 33 are involved in the formation of intermediate diphosphate precursors via the MEP and MVA pathways, five are members of the *SmCPS* gene families, while the other two encode SmKSLs. Analysis of GA metabolism and regulation may greatly help to demonstrate the role of GAs in *S. miltiorrhiza* growth and development and the crosstalk between GA metabolism and tanshinone biosynthesis, which are very important for genetic improvement of *S. miltiorrhiza*. However, genes involved in the conversion of *ent*-kaurene to GAs have not been systematically studied. In this study, genome-wide identification, molecular cloning and expression analysis of the *SmKO*, *SmKAO*, *SmGA20ox*, *SmGA3ox* and *SmGA2ox* gene families in *S. miltiorrhiza* were carried out. The results identified tissue-specifically expressed, feedback-regulated, stress-responsive and alternatively spliced novel genes, revealed multiple layer regulation of GA metabolism, and provided evidence for crosstalk between GA metabolism and tanshinone biosynthesis in *S. miltiorrhiza*.

## Results

### Prediction and molecular cloning of GA metabolism pathway genes in *S. miltiorrhiza*

In order to predict GA metabolism pathway genes in *S. miltiorrhiza*, we downloaded the deduced amino acid sequences of 19 *Arabidopsis* and 24 rice KO, KAO, GA2ox, GA3ox and GA20ox proteins from the *Arabidopsis* Information Resource (TAIR, http://www.arabidopsis.org) and the China Rice Data Center (http://www.ricedata.cn/gene), respectively. BLAST analysis of the downloaded *Arabidopsis* and rice sequences against the current assembly of the *S. miltiorrhiza* genome was then performed using the tBLASTn algorithm [39, 40]. The retrieved *S. miltiorrhiza* genomic DNA sequences putatively encoding proteins with more than 50 % identity to *Arabidopsis* or rice homologs were predicted for gene models on the Genscan web server (http://genes.mit.edu/GENSCAN.html) [41] and the NCBI BLAST (http://blast.ncbi.nlm.nih.gov/Blast.cgi) [40]. As a result, 22 gene models, including one for *SmKO*, two for *SmKAOs*, six for *SmGA20oxs*, two for *SmGA3oxs* and eleven for *SmGA2oxs*, were predicted. Among them, sixteen putatively encode full-length proteins, while the other six are partial.

In order to verify the predicted gene models and to obtain full-length coding sequences, molecular cloning was carried out using PCR techniques. The results showed that all of the predicted gene models were experimentally validated and full-length coding sequences of the 22 predicted genes were obtained (Table 1). Based on sequence similarities between the cloned cDNAs and the known genes in other plant species, the identified genes were named *SmKO*, *SmKAO1*, *SmKAO2*, *SmGA20ox1*–*SmGA20ox6*, *SmGA3ox1*, *SmGA3ox2*, and *SmGA2ox1*–*SmGA2ox11*, respectively. The cloned nucleotide sequences and their deduced amino acid sequences may be found in GenBank under the accession numbers shown in Table 1.

**Table 1:** Sequence features of GA metabolism pathway genes in *S. miltiorrhiza*

Gene name	Accession number	ORF^a^ (bp)	Len^b^	MW^c^ (kDa)	p*I*	Loc^d^
*SmKO*	KT853074	1557	519	58.73	7.63	S
*SmKAO1*	KT853075	1302	434	50.00	9.33	S
*SmKAO2*	KT853076	1431	477	54.82	9.43	S
*SmGA3ox1*	KT853077	1038	346	37.86	6.70	-
*SmGA3ox2*	KT853078	1092	364	39.92	7.74	-
*SmGA20ox1*	KT853079	1134	378	43.01	6.38	-
*SmGA20ox2*	KT853080	1173	391	43.83	6.56	-
*SmGA20ox3*	KT853081	1155	385	43.01	7.69	-
*SmGA20ox4*	KT853082	1176	392	44.09	6.11	C
*SmGA20ox5*	KT853083	1044	348	38.50	5.99	-
*SmGA20ox6*	KT853084	1050	350	39.99	5.84	-
*SmGA2ox1*	KT853085	1122	374	42.12	7.54	C
*SmGA2ox2*	KT853086	966	322	35.70	5.60	-
*SmGA2ox3*	KT853087	936	312	34.66	6.12	-
*SmGA2ox4*	KT853088	1005	335	37.67	6.22	-
*SmGA2ox5*	KT853089	1002	334	38.15	7.29	C
*SmGA2ox6*	KT853090	987	329	37.39	5.56	-
*SmGA2ox7*	KT853091	939	313	35.16	5.38	-
*SmGA2ox8*	KT853092	1005	335	37.19	7.08	-
*SmGA2ox9*	KT853093	933	311	34.36	5.85	-
*SmGA2ox10*	KT853094	1137	379	41.83	5.69	-
*SmGA2ox11*	KT853095	972	324	36.01	6.67	-

^a^ORF represents open reading frame

^b^Len represents the number of amino acid residues

^c^MW represents molecular weight

^d^Loc represents the protein localization predicted by TargetP1.1. ‘S’ stands for secretory pathway, showing that the sequence cotains a signal peptide. ‘C’ stands for chloroplast, suggesting that the sequence contains a chloroplast transit peptide. ‘-’ indicates any locations other than the plastid, mitochondrion and secretory pathway

BLAST analysis of the *Arabidopsis*, rice and *S. miltiorrhiza* sequences against the *S. miltiorrhiza* transcriptomic unigenes assembled from ESTs and RNA-seq reads [42] showed that unigenes could be identified for nine of the 22 cloned *S. miltiorrhiza* GA metabolism pathway genes. It includes *SmKO*, *SmKAO1*, *SmGA3ox2*, *SmGA20ox2*–*SmGA20ox4*, *SmGA2ox3*, *SmGA2ox5* and *SmGA2ox11*. No additional GA metabolism pathway genes were identified. BLAST analysis of the cloned cDNAs against the non-redundant protein sequence (nr) database (http://blast.ncbi.nlm.nih.gov/Blast.cgi) using the BLASTn algorithm with default parameters [40] showed that the coding regions of *SmKO* and *SmKAO1* had just been identified through the analysis of high-throughput RNA-Seq data and reported as CYP701A40 (accession no. KP337739) and CYP88A52 (KP337715), respectively [43]. Additionally, *SmKO* (KJ606394) was also cloned from the hairy roots of *S. miltiorrhiza* (line Shanxishangluo) [44]. It further verifies our results from computational prediction and experimental cloning.

### Characterization and expression analysis of *SmKO*

**Fig. 1:**
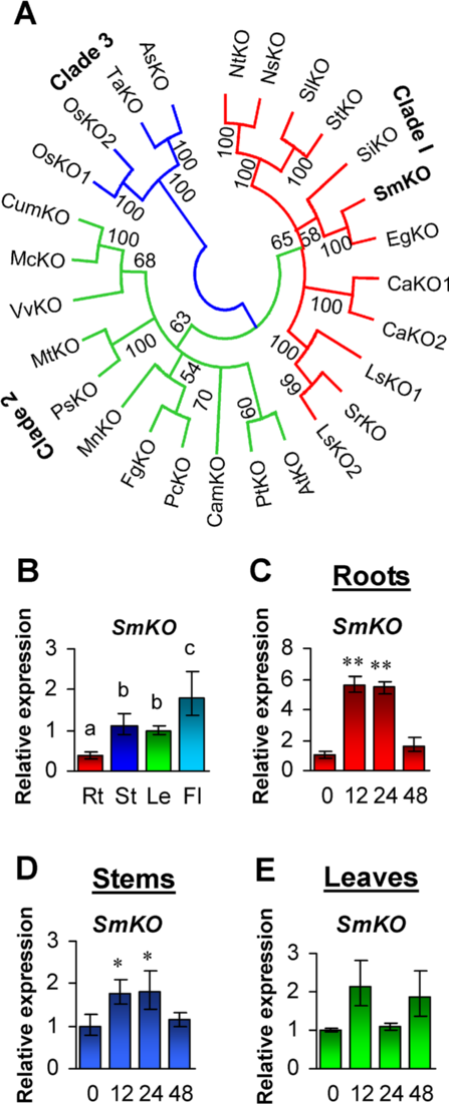
Phylogenetic analysis of KOs and expression profiling of *SmKO*. **a**: Phylogenetic relationship of KOs in *Aegilops speltoides* (As), *Arabidopsis thaliana* (At), *Castanea mollissima* (Cam), *Coffea arabica* (Ca), *Cucurbita maxima* (Cum), *Erythranthe guttata* (Eg), *Fragaria grandiflora* (Fg), *Lactuca sativa* (Ls), *Medicago truncatula* (Mt), *Momordica charantia* (Mc), *Morus notabilis* (Mn), *Nicotiana sylvestris* (Ns), *Nicotiana tomentosiformis* (Nt), *Oryza sativa* (Os), *Pisum sativum* (Ps), *Populus trichocarpa* (Pt), *Pyrus communis* (Pc), *S. miltiorrhiza* (Sm), *Sesamum indicum* (Si), *Solanum lycopersicum* (Sl), *Solanum tuberosum* (St), *Stevia rebaudiana* (Sr), *Triticum aestivum* (Ta), and *Vitis vinifera*(Vv). The unrooted neighbor-joining tree was constructed using the MEGA 6.0 [64]. SmKO from *S. miltiorrhiza* are in bold. Clades 1–3 indicate the three clades identified. **b**: Fold changes of *SmKO* genes in roots (Rt), stems (St), leaves (Le) and flowers (Fl) of *S. miltiorrhiza* plants. The expression levels were analyzed using the quantitative RT-PCR method. Expression level in leaves was arbitrarily set to 1 and the levels in other tissues were given relative to this. Error bars represent standard deviations of mean value from three biological and four technical replicates. ANOVA (analysis of variance) was calculated using SPSS. *P* < 0.05 was considered statistically significant. **c**–**e**: Responses of *SmKO* to exogenous GA3 treatment. Fold changes of *SmKO* transcripts in roots (**c**), stems (**d**) and leaves (**e**) of *S. miltiorrhiza* plantlets treated with 100 μM GA3 for 0, 12, 24 and 48 h are shown. The expression levels were analyzed using the quantitative RT-PCR method. Expression level in tissues without treatment (0 h) was arbitrarily set to 1 and the levels in tissues from GA3-treated plantlets were given relative to this. Error bars represent standard deviations of mean value from three biological and four technical replicates. ANOVA (analysis of variance) was calculated using SPSS. *P* < 0.05 (*) and *P* < 0.01 (**) was considered statistically significant and extremely significant, respectively

KO catalyzes the conversion of *ent*-kaurene to *ent*-kaurenoic acid via *ent*-kaurenol and *ent*-kaurenal. The cloned *SmKO* cDNA encodes a protein with the amino acid number of 519, the theoretical isoelectric point (p*I*) of 7.63, and the predicted molecular weight (MW) of 58.73 kDa (Table 1). It shares over 60 % identities with KOs in various other plants, such as *Sesamum indicum* (XP_011083784) and *Erythranthe guttata* (EYU45074). Phylogenetic analysis of 27 KOs from 24 plant species showed that plant KOs could be divided into three clades (Fig. 1). SmKO clusters with EgKO, SiKO and other nine KOs from seven species in clade 1, AtKO clusters with PtKO and other ten KOs in clade 2, while OsKO1 and OsKO2 clusters with TaKO and AsKO in clade 3 (Fig. 1a). The results indicate that the cloned *SmKO* encodes a *bona fide ent*-kaurene oxidase in *S. miltiorrhiza*.

Like other P450 proteins, SmKO contains the P450 conserved domain (Additional file 1: Figure S1). Protein subcellular localization prediction indicates that SmKO contains a 15 bp secretory pathway signal peptide at the N-terminus, suggesting this protein is most possibly present in the endoplasmic reticulum (Table 1). Similar results were also obtained for *Arabidopsis* and rice KOs, including AtKO, OsKO1 and OsKO2 (Table 1, Additional files 2 and 3: Tables S1 and S2), indicating that the conversion of *ent*-kaurene to *ent*-kaurenoic acid is associated with the endoplasmic reticulum.

In consistence with the role of GA in plant growth and development, *SmKO* showed ubiquitous expression in all the tissues analyzed, with the highest in flowers, followed by stems and leaves, and the lowest in roots (Fig. 1b). The expression pattern of *SmKO* is similar to that of *AtKO* showing the highest level in inflorescence, less in elongating stems, and relatively low in both rosette and cauline leaves [45]. In order to know the response of GA metabolism pathway genes to active GAs in *S. miltiorrhiza*, we analyzed the expression of *SmKO* in roots, stems and leaves of *S. miltiorrhiza* treated with exogenous 100 μM GA_3_ for 12, 24 and 48 h, respectively. Relative expression was analyzed using the qRT-PCR method as described previously [12]. The results showed that *SmKO* was up-regulated in roots and stems of *S. miltiorrhiza* plants treated with exogenous GA_3_ for 12 and 24 h (Fig. 1c and 1d). No significant changes were found in leaves at all three time-points of GA_3_ treatment and in roots and stems at the time-point of 48-h-treatment (Fig. 1c–1e), suggesting tissue-specificity of *SmKO* response to GA_3_ treatment.

### Characterization and expression analysis of *SmKAO1* and *SmKAO2*

**Fig. 2:**
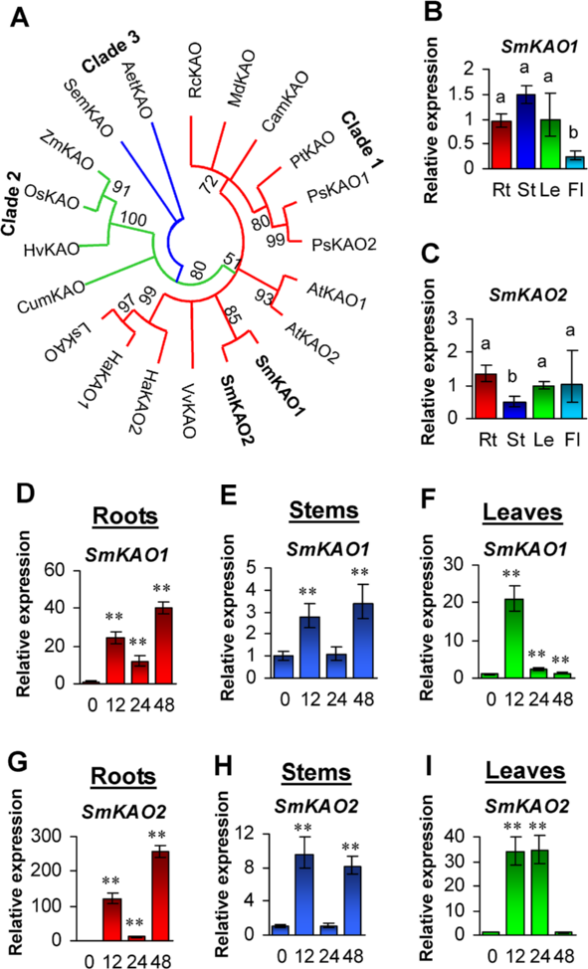
Phylogenetic analysis of KAOs and expression profiling of *SmKAO1* and *SmKAO2*. **a**: Phylogenetic relationship of KAOs in *Aegilops tauschii* (Aet), *Arabidopsis thaliana* (At), *Castanea mollissima* (Cam), *Cucurbita maxima* (Cum), *Helianthus annuus* (Ha), *Hordeum vulgare* (Hv), *Lactuca sativa* (Ls), *Malus domestica* (Md), *Oryza sativa* (Os), *Pisum sativum* (Ps), *Populus trichocarpa* (Pt), *Ricinus communis* (Rc), *S. miltiorrhiza* (Sm), *Selaginella moellendorffii* (Sem), *Vitis vinifera*(Vv), and *Zea mays* (Zm). The unrooted neighbor-joining tree was constructed using the MEGA 6.0 [64]. SmKAOs from *S. miltiorrhiza* are in bold. Clades 1–3 indicate the three clades identified. **b** and **c**: Fold changes of *SmKAO1* (**b**) and *SmKAO2* (**c**) genes in roots (Rt), stems (St), leaves (Le) and flowers (Fl) of *S. miltiorrhiza* plants. The expression levels were analyzed using the quantitative RT-PCR method. Expression level in leaves was arbitrarily set to 1 and the levels in other tissues were given relative to this. Error bars represent standard deviations of mean value from three biological and four technical replicates. ANOVA (analysis of variance) was calculated using SPSS. *P* < 0.05 was considered statistically significant. **d**–**i**: Responses of *SmKAOs* to exogenous GA3 treatment. Fold changes of *SmKAO1* (**d**–**f**) and *SmKAO2* (**g**–**i**) transcripts in roots, stems and leaves of *S. miltiorrhiza* plantlets treated with 100 μM GA3 for 0, 12, 24 and 48 h are shown. The expression levels were analyzed using the quantitative RT-PCR method. Expression level in tissues without treatment (0 h) was arbitrarily set to 1 and the levels in tissues from GA3-treated plantlets were given relative to this. Error bars represent standard deviations of mean value from three biological and four technical replicates. ANOVA (analysis of variance) was calculated using SPSS. *P* < 0.05 (*) and *P* < 0.01 (**) was considered statistically significant and extremely significant, respectively

KAO catalyzes the conversion of *ent*-kaurenoic acid to GA_12_ via *ent*-7α-hydroxykaurenoic acid and GA_12_-aldehyde. One *OsKAO* and two *AtKAO* genes were identified in rice and *Arabidopsis*, respectively [16, 25]. From *S. miltiorrhiza*, we identified two *KAO* genes, *SmKAO1* and *SmKAO2. SmKAO1* encodes a protein with 434 amino acid residues, while *SmKAO* encodes a protein consisting of 477 amino acid residues (Table 1). Both of them contain the P450 domain. It is consistent with previous results showing that KAOs are members of the P450 family [29]. SmKAO1 and SmKAO2 share about 58 % identity at the amino acid level and are clustered together in the phylogenetic tree constructed with 15 KAOs from 11 plant species (Fig. 2a). Consistently, two KAOs from other plant species, such as *Arabidopsis*, *Helianthus annuus* and *Pisum sativum*, are also clustered together in the phylogenetic tree (Fig. 2a). It suggests that two KAOs from a plant species are usually paralogous proteins. *SmKAO1* and *SmKAO2* were expressed in all tissues examined (Fig. 2b and 2c). The expression of *SmKAO1* was higher in stems, roots and leaves than flowers, while the expression of *SmKAO2* was higher in roots, flowers and leaves and less in stems (Fig. 2b and 2c). Analysis of *SmKAOs* to exogenous GA_3_ showed that *SmKAO1* was up-regulated in roots and leaves at all three time-points of GA_3_ treatment and in stems at the time-points of 12- and 48-h-treatement (Fig. 2d–2f). *SmKAO2* was up-regulated in roots at all three time-points of GA_3_ treatment, in stems at the time-points of 12- and 48-h-treatement, and in leaves treated with GA_3_ for 12 and 24 h (Fig. 2g–2i). It suggests differential responses of *SmKAO1* and *SmKAO2* to GA_3_ treatment.

### Characterization and expression analysis of the *SmGA20ox* gene family

GA20ox represents an important regulatory node in GA metabolism and plays significant roles in maintaining the endogenous GA level in plants [20, 46, 47]. It catalyzes the conversion of GA_12_ and GA_53_ (C_20_-GAs) to GA_9_ and GA_20_ (C_19_-GAs), respectively. The reaction requires successive oxidation of C-20 from the methyl group of C_20_-GAs through the alcohol and then aldehyde, from which the C-20 is lost as carbon dioxide [13]. Usually, a single enzyme with full GA20ox catalytic activity can catalyze this reaction sequence [13]. However, few GA20ox enzymes catalyze only partial of the reaction sequence. For instance, there are a total of five *Arabidopsis* AtGA20ox enzymes, of which, four, including AtGA20ox1–AtGA20ox4, possess full GA20ox activity in vitro, while the other one, named AtGA20ox5, can only catalyze the first two steps of the reaction sequence [18, 20].

**Fig. 3:**
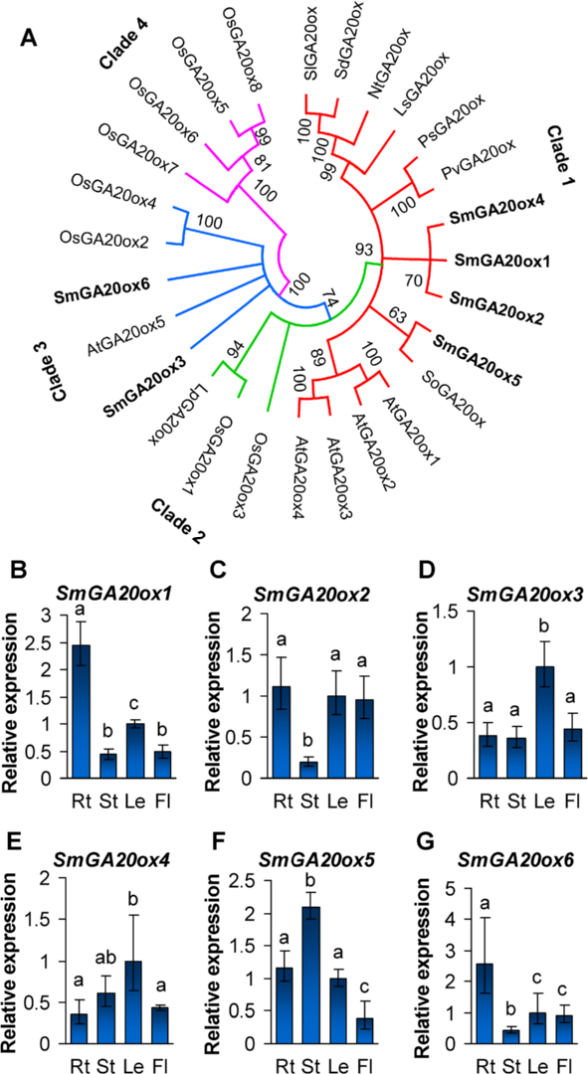
Phylogenetic analysis of GA20ox proteins and expression profiling of *SmGA20ox* genes. **a**: Phylogentic relationship of GA20ox proteins in *Arabidopsis thaliana* (At), *Lactuca sativa* (Ls), *Lolium perenne* (Lp), *Nicotiana tabacum* (Nt), *Oryza sativa* (Os), *Phaseolus vulgaris* (Pv), *Pisum sativum* (Ps), *Salvia miltiorrhiza* (Sm), *Solanum dulcamara* (Sd), *Solanum lycopersicum* (Sl), and *Spinacia oleracea* (So). The unrooted neighbor-joining tree was constructed using the MEGA 6.0 [64]. SmGA20ox proteins from *S. miltiorrhiza* are in bold. Clades 1–4 indicate the four clades identified. **b**–**g**: Fold changes of *SmGA20ox1* (**b**), *SmGA20ox2* (**c**), *SmGA20ox3* (**d**), *SmGA20ox4* (**e**), *SmGA20ox5* (**f**) and *SmGA20ox6* (**g**) in roots (Rt), stems (St), leaves (Le) and flowers (Fl) of *S. miltiorrhiza* plants. The expression levels were analyzed using the quantitative RT-PCR method. Expression level in leaves was arbitrarily set to 1 and the levels in other tissues were given relative to this. Error bars represent standard deviations of mean value from three biological and four technical replicates. ANOVA (analysis of variance) was calculated using SPSS. *P* < 0.05 was considered statistically significant

From *S. miltiorrhiza*, a total of six *SmGA20ox* genes were identified. All of them contain the DIOX_N and 2OG-FeII_Oxy domains found in 2-oxoglutarate/Fe(II)-dependent dioxygenases (Additional file 1: Figure S1). Sequence comparison showed that the six SmGA20ox proteins could be roughly divided into two groups. SmGA20ox1, SmGA20ox2, SmGA20ox4 and SmGA20ox5 share over 60 % sequence identity in a group, while SmGA20ox3 and SmGA20ox6 share about 50 % identity in the other group. Phylogenetic analysis of 27 GA20ox proteins from *Arabidopsis*, rice, and other eight plant species showed that plant GA20ox proteins could be divided into four clades (Fig. 3a). SmGA20ox1, SmGA20ox2, SmGA20ox4 and SmGA20ox5 group with AtGA20ox1–AtGA20ox4 and other seven GA20ox proteins from various plant species in clade 1. SmGA20ox3 and SmGA20ox6 cluster with AtGA20ox5, OsGA20ox2 and OsGA20ox4 in clade 3. Clade 2 and clade 4 mainly include GA20ox proteins from rice. No SmGA20ox and AtGA20ox proteins belong to the two clades. It indicates the conservation and divergence of GA20ox proteins in plants.

Since the majority of GA20ox proteins showed full catalytic activity in converting C_20_ substrates to C_19_ products, the expression patterns, rather than enzymatic activities, determine their physiological roles in plants [20]. Using the qRT-PCR method, we analyzed the expression patterns of 6 *SmGA20ox* genes in roots, stems, leaves and flowers of *S. miltiorrhiza* plants. Obvious differential expression of *SmGA20ox* genes was observed. *SmGA20ox1* were predominantly expressed in roots, followed by leaves, and the lowest in stems and flowers (Fig. 3b). *SmGA20ox2* showed high expression in roots, leaves and flowers, while its expression in stems was significantly low (Fig. 3c). *SmGA20ox3* exhibited the highest expression in leaves, less in stems, flowers and roots (Fig. 3d). *SmGA20ox4* showed high expression in leaves and stems, less in roots and flowers (Fig. 3e). *SmGA20ox5* exhibited the highest expression in stems, followed by roots and leaves, and the lowest in flowers (Fig. 3f). *SmGA20ox6* had the highest expression in roots, followed by leaves and flowers, and the lowest in stems (Fig. 3g). Differential expression of *GA20ox* genes was also observed in other plant species, such as *Arabidopsis* [20], rice [25] and maize [28], indicating that different members of the *GA20ox* gene family might play distinct physiological roles. Since the transcripts of multiple *SmGA20ox* genes exist in a tissue, it is possible that some of them act redundantly in plant growth and development.

**Fig. 4:**
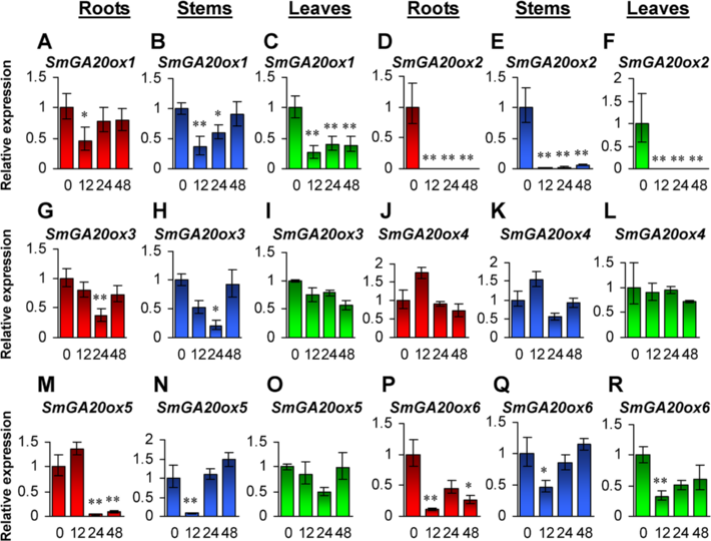
Responses of *SmGA20ox* genes to exogenous GA3 treatment. Fold changes of *SmGA20ox1* (**a**–**c**), *SmGA20ox2* (**d**–**f**), *SmGA20ox3* (**g**–**i**), *SmGA20ox4* (**j**–**l**), *SmGA20ox5* (**m**–**o**) and *SmGA20ox6* (**p**–**r**) in roots, stems and leaves of *S. miltiorrhiza* plantlets treated with 100 μM GA3 for 0, 12, 24 and 48 h are shown. The expression levels were analyzed using the quantitative RT-PCR method. Expression level in tissues without treatment (0 h) was arbitrarily set to 1 and the levels in tissues from GA3-treated plantlets were given relative to this. Error bars represent standard deviations of mean value from three biological and four technical replicates. ANOVA (analysis of variance) was calculated using SPSS. *P* < 0.05 (*) and *P* < 0.01 (**) was considered statistically significant and extremely significant, respectively

Consistently, among the six *SmGA20ox* genes, *SmGA20ox2* was significantly down-regulated in roots, stems and leaves treated with GA_3_ for 12, 24 and 48 h (Fig. 4d–4f). Down-regulated expression was also found for *SmGA20ox1* in roots at the time-point of 12-h-treatment, in stems at the time-points of 12- and 24-h-treatment, and leaves at all three time-points (Fig. 4a–4c). Similarly, *SmGA20ox3* was down-regulated in roots and stems at the time-point of 24-h-treatment (Fig. 4g and 4h). *SmGA20ox5* was down-regulated in roots at the time-points of 24- and 48-h-treatment (Fig. 4m and 4n). *SmGA20ox6* was down-regulated in roots at the time-point of 12- and 48-h-treatment, and in stems and leaves at the time-point of 12-h-treatment (Fig. 4p–4r). It suggests the existence of negative feedback mechanism to regulate the expression of some *SmGA20ox* genes in *S. miltiorrhiza*. No significant changes were observed for the expression of *SmGA20ox4* in roots, stems and leaves (Fig. 4j–4l), *SmGA20ox3* and *SmGA20ox5* in leaves (Fig. 4i and 4o), and *SmGA20ox1* (Fig. 4a and 4b), *SmGA20ox3* (Fig. 4g and 4h), *SmGA20ox5* (Fig. 4m and 4n), *SmGA20ox6* (Fig. 4p and 4q) in roots and leaves at one or two time-points of GA_3_ treatment, suggesting that the responses of *SmGA20* genes to GA_3_ treatment depend on tissue types and time-points of GA_3_ treatment in *S. miltiorrhiza*.

### Characterization and expression analysis of *SmGA3ox1* and *SmGA3ox2*

GA3ox plays a direct role in conversion of precursor GAs to their biologically active forms and determines the level of bioactive GAs in plants [19]. It catalyzes the formation of final bioactive GA_4_ and GA_1_ from GA_9_ and GA_20_, respectively, in a single step in plants. It also catalyzes the production of small amounts of GA_3_, a C-1,2-unsaturated bioactive GA, in a two-step reaction via GA_5_ in several monocotyledons [4]. From the *S. miltiorrhiza* genome, we identified and cloned two *SmGA3ox* genes, named *SmGA3ox1* and *SmGA3ox2*, respectively (Table 1). *SmGA3ox1* contains 1038 bp of ORF encoding a protein with 346 amino acids, while *SmGA3ox2* contains 1092 bp of ORF encoding a protein with 364 amino acids.

**Fig. 5:**
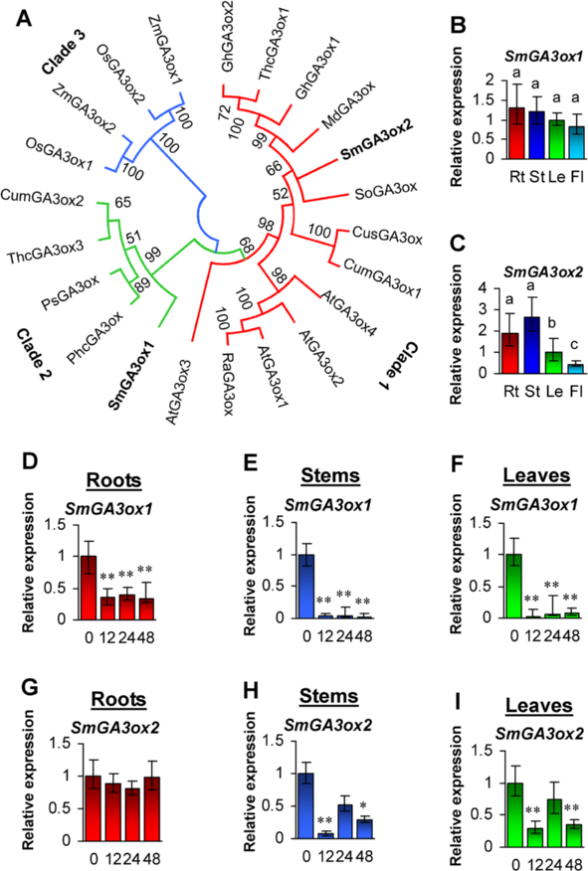
Phylogenetic analysis of GA3ox proteins and expression profiling of *SmGA3ox* genes. **a**: Phylogentic relationship of GA3ox proteins in *Arabidopsis thaliana* (At), *Cucumis sativus* (Cus), *Cucurbita maxima* (Cum), *Gossypium hirsutum* (Gh), *Malus domestica* (Md), *Oryza sativa* (Os), *Physcomitrella patens* (Pp), *Pisum sativum* (Ps), *Rorippa aquatica* (Ra), *Salvia miltiorrhiza* (Sm), *Spinacia oleracea* (So), *Theobroma cacao* (Thc), and *Zea mays* (Zm). The unrooted neighbor-joining tree was constructed using the MEGA 6.0 [64]. SmGA3ox1 and SmGA3ox2 from *S. miltiorrhiza* are in bold. Clades 1–3 indicate the three clades identified. **b** and **c**: Fold changes of *SmGA3ox1* (**b**) and *SmGA3ox2* (**c**) in roots (Rt), stems (St), leaves (Le) and flowers (Fl) of *S. miltiorrhiza* plants. The expression levels were analyzed using the quantitative RT-PCR method. Expression level in leaves was arbitrarily set to 1 and the levels in other tissues were given relative to this. Error bars represent standard deviations of mean value from three biological and four technical replicates. ANOVA (analysis of variance) was calculated using SPSS. *P* < 0.05 was considered statistically significant. **d**–**i**: Responses of *SmGA3ox* genes to exogenous GA3 treatment. Fold changes of *SmGA3ox1* (**d**–**f**) and *SmGA3ox2* (**g**–**i**) transcripts in roots, stems and leaves of *S. miltiorrhiza* plantlets treated with 100 μM GA3 for 0, 12, 24 and 48 h are shown. The expression levels were analyzed using the quantitative RT-PCR method. Expression level in tissues without treatment (0 h) was arbitrarily set to 1 and the levels in tissues from GA3-treated plantlets were given relative to this. Error bars represent standard deviations of mean value from three biological and four technical replicates. ANOVA (analysis of variance) was calculated using SPSS. *P* < 0.05 (*) and *P* < 0.01 (**) was considered statistically significant and extremely significant, respectively

Phylogenetic analysis of 22 GA3ox proteins from 13 plant species showed that plant GA3ox could be divided into 3 clades (Fig. 5a). SmGA3ox1 and SmGA3ox2 were separated into two clades (Fig. 5a), suggesting the divergence of SmGA3ox1 and SmGA3ox2. Consistently, differential expression was observed between *SmGA3ox1* and *SmGA3ox2* (Fig. 5b and 5c). *SmGA3ox1* expressed broadly in the tissues analyzed, while *SmGA3ox2* exhibited more tissue-specific expression with the highest level in stems and roots, followed by leaves, and the lowest in flowers. Differential expression was also observed for *Arabidopsis* and rice *GA3ox* genes [19, 21, 25]. Of the two rice *OsGA3ox* genes, *OsGA3ox1* was preferentially expressed in the panicles, while *OsGA3ox2* was broadly expressed in all organs tested [25]. Among the four *AtGA3ox* genes, *AtGA3ox1* was expressed throughout development; *AtGA3ox2* was mainly expressed during seed germination and vegetative growth; while *AtGA3ox3* and *AtGA3ox4* were predominantly expressed in reproductive organs [19, 21]. Thus, *SmGA3ox1* appears to be important for the development of both vegetative and reproductive organs, while *SmGA3ox2* is more specific to bioactive GA biosynthesis during vegetative growth of *S. miltiorrhiza*. Analysis of *SmGA3oxs* to exogenous GA_3_ showed that *SmGA3ox1* was down-regulated in roots, stems and leaves treated with GA_3_ for 12, 24 and 48 h (Fig. 5d–5f). *SmGA3ox2* was down-regulated in stems and leaves of *S. miltiorrhiza* treated with GA_3_ for 12 and 48 h (Fig. 5h and 5i). No changes were found for *SmGA3ox2* in roots at all three time-points and in stems and leaves at the time-point of 24-h-treatment (Fig. 5g–5i). It suggests that each member of the *GA3ox* gene family from a plant species may play different physiological roles.

### Characterization and expression analysis of the *SmGA2ox* gene family

Contrary to GA3ox involved in bioactive GA formation, GA2ox catalyzes the deactivation of bioactive GAs, such as GA_1_ and GA_4_, by introducing a 2β-hydroxy group to the GAs [15, 22, 23]. In addition to the bioactive C_19_-GAs, GA2ox can also use C_19_- (GA_9_ and GA_20_) and C_20_-GA precursors (GA_12_ and GA_53_) of bioactive GAs as substrates. Therefore, GA2ox is important for turnover of the physiologically active GAs, allowing precise regulation of GA concentration in plant tissues [4, 13]. GA2ox is usually encoded by a small gene family in plants. Based on the substrates, GA2ox proteins can be divided into two groups, one group, known as C_19_-GA2ox proteins, using bioactive C_19_-GAs and C_19_-GA precursors as substrates, while the other group, known as C_20_-GA2ox, acting on C_20_-GA precursors [15, 22, 23].

**Fig. 6:**
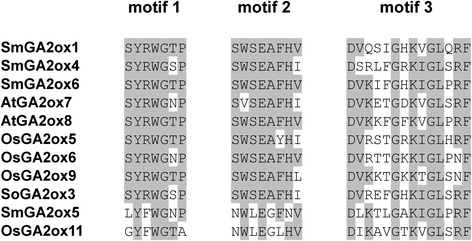
Sequence alignment of the three unique and conserved motifs identified in various GA2ox proteins. *A. thaliana* (At), *O. sativa* (Os), *Spinacia oleracea* (So) and *S. miltiorrhiza* (Sm)

From the current assembly of the *S. miltiorrhiza* genome, we predicted 11 *SmGA2ox* genes (Table 1). The prediction was verified by PCR amplification and subsequent sequencing. All of the deduced SmGA2ox proteins contain the conserved domains, DIOX_N and 2OG-FeII_Oxy (Additional file 1: Figure S1). It is consistent with their 2-oxoglutarate/Fe(II)-dependent dioxygenase activity. Moreover, SmGA2ox1, SmGA2ox4 and SmGA2ox6 contain the three unique and conserved motifs (Fig. 6) identified in AtGA2ox7, AtGA2ox8, OsGA2ox5, OsGA2ox6, OsGA2ox9 and SoGA2ox3, which use C_20_-GA precursors as substrates [22, 23]. It indicates that SmGA2ox1, SmGA2ox4 and SmGA2ox6 are C_20_-GA2ox proteins. Interestingly, SmGA2ox5 also contain the three motifs; however, the sequences of these motifs are less conserved compared with those in SmGA2ox1, SmGA2ox4 and SmGA2ox6 (Fig. 6). Further analysis of the motifs in AtGA2ox and OsGA2ox proteins showed that OsGA2ox11 also contained the three less conserved motifs (Fig. 6). It implies that SmGA2ox5 and OsGA2ox11 may be C_20_-GA2ox proteins with specific functions. The three motifs are absent from other seven SmGA2ox proteins, such as SmGA2ox2, SmGA2ox3, SmGA2ox7–SmGA2ox11, indicating they are C_19_-GA2ox proteins.

**Fig. 7:**
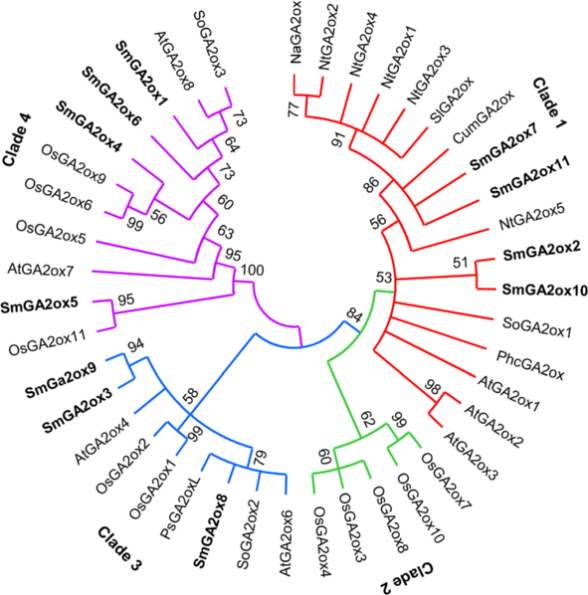
Phylogenetic analysis of GA2ox proteins in *S. miltiorrhiza* and various other plant species. It includes *Arabidopsis thaliana* (At), *Cucurbita maxima* (Cum), *Nicotiana attenuata* (Na), *Nicotiana tabacum* (Nt), *Oryza sativa* (Os), *Phaseolus coccineus* (Phc), *Pisum sativum* (Ps), *Salvia miltiorrhiza* (Sm), *Solanum lycopersicum* (Sl), and *Spinacia oleracea* (So). The unrooted neighbor-joining tree was constructed using the MEGA 6.0 [64]. SmGA2ox proteins from *S. miltiorrhiza* are in bold. Clades 1–4 indicate the four clades identified

Phylogenetic relationship among 42 GA2ox proteins from 10 plant species was analyzed. Based on the phylogenetic tree constructed, plant GA2ox proteins could be divided into four clades (Fig. 7). SmGA2ox2, SmGA2ox7, SmGA2ox10, SmGA2ox11, AtGA2ox1–AtGA2ox3 and other ten plant GA2ox proteins group in clade 1. Five rice OsGA2ox proteins, including OsGA2ox3, OsGA2ox4, OsGA2ox7, OsGA2ox8 and OsGA2ox10, cluster in clade 2. SmGA2ox3, SmGA2ox8, SmGA2ox9, AtGA2ox4, AtGA2ox6, OsGA2ox1, OsGA2ox2, SoGA2ox2 and PsGA2oxL group in clade 3. The other eleven, including SmGA2ox1, SmGA2ox4, SmGA2ox5, SmGA2ox6 and OsGA2ox11 and the known C_20_-GA2ox proteins (AtGA2ox7, AtGA2ox8, OsGA2ox5, OsGA2ox6, OsGA2ox9 and SoGA2ox3), cluster in clade 4. It is similar with previous results from plant GA2ox analysis [22, 23], suggesting members of clades 1, 2 and 3 are C_19_-GA2ox proteins, while members of clade 4 are C_20_-GA2ox proteins.

**Fig. 8:**
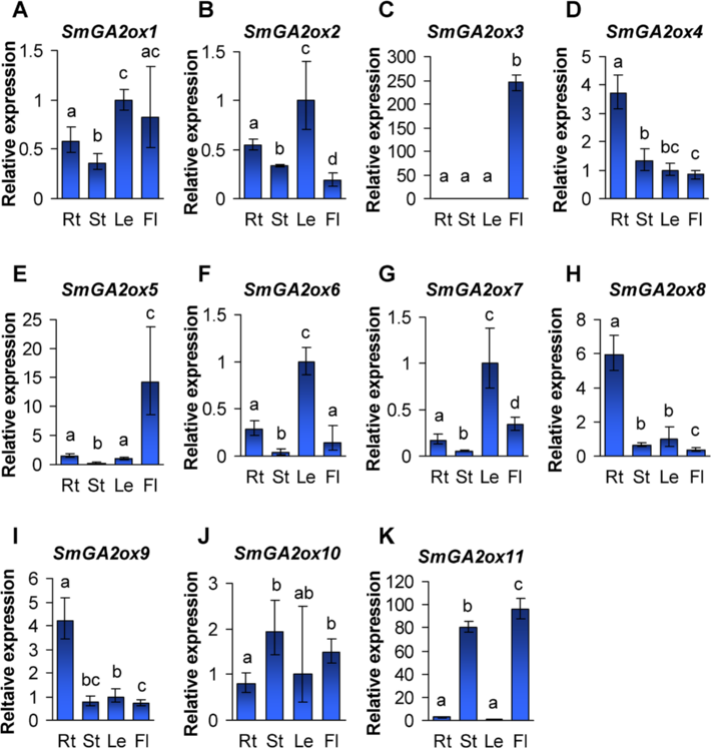
Expression patterns of *SmGA2ox* genes in *S. miltiorrhiza* plants. **a**–**k**: Fold changes of *SmGA2ox1* (**a**), *SmGA2ox2* (**b**), *SmGA2ox3* (**c**), *SmGA2ox4* (**d**), *SmGA2ox5* (**e**), *SmGA2ox6* (**f**), *SmGA2ox7* (**g**), *SmGA2ox8* (**h**), *SmGA2ox9* (**i**), *SmGA2ox10* (**j**) and *SmGA2ox11* (**k**) genes in roots (Rt), stems (St), leaves (Le) and flowers (Fl) of *S. miltiorrhiza* plants. The expression levels were analyzed using the quantitative RT-PCR method. Expression level in leaves was arbitrarily set to 1 and the levels in other tissues were given relative to this. Error bars represent standard deviations of mean value from three biological and four technical replicates. ANOVA (analysis of variance) was calculated using SPSS. *P* < 0.05 was considered statistically significant

To investigate the expression pattern of each *SmGA2ox* gene, we analyzed their transcript levels in roots, stems, leaves and flowers of *S. miltiorrhiza* using the qRT-PCR method. Similarly with the results from *SmGA20ox* and *SmGA3ox* genes, *SmGA2ox* genes showed obvious differential expression (Fig. 8). *SmGA2ox4*, *SmGA2ox8* and *SmGA2ox9* were mainly expressed in roots (Fig. 8d, 8h and 8i). *SmGA2ox2*, *SmGA2ox6* and *SmGA2ox7* showed the highest expression in leaves, followed by roots, flowers and stems (Fig. 8b, 8f and 8g). *SmGA2ox3* and *SmGA2ox5* were predominantly expressed in flowers (Fig. 8c and 8e). *SmGA2ox1* showed the highest expression in leaves and flowers, followed by roots and stems (Fig. 8a). *SmGA2ox10* exhibited the highest expression in stems, followed by flowers, leaves and roots (Fig. 8j). *SmGA2ox11* was predominantly expressed in flowers and stems (Fig. 8k). Distinct expression patterns were also observed for members of the *GA2ox* gene family in *Arabidopsis* [15] and rice [23, 25]. These results suggest the redundant and diversified physiological roles of *GA2ox* genes played in plants.

**Fig. 9:**
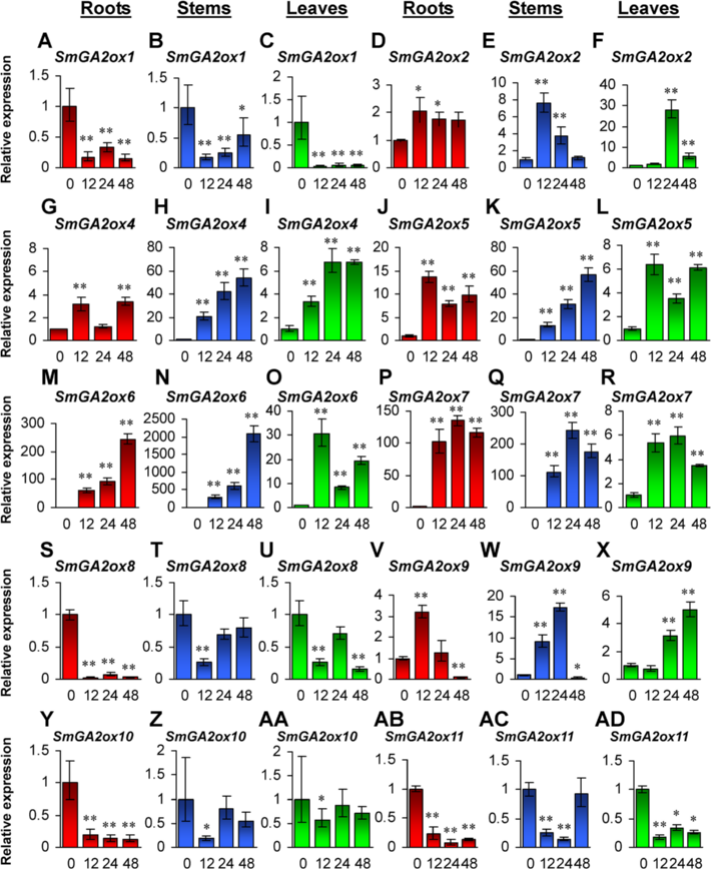
Responses of *SmGA2ox* genes to exogenous GA3 treatment. **a**–**ad**: Fold changes of *SmGA2ox1* (**a**–**c**), *SmGA2ox2* (**d**–**f**), *SmGA2ox4* (**g**–**i**), *SmGA20ox5* (**j**–**l**), *SmGA20ox6* (**m**–**o**), *SmGA20ox7* (**p**–**r**), *SmGA2ox8* (**s**–**u**), *SmGA2ox9* (**v**–**x**), *SmGA2ox10* (**y**–**aa**) and *SmGA2ox11* (**ab**–**ad**) in roots, stems and leaves of *S. miltiorrhiza* plantlets treated with 100 μM GA3 for 0, 12, 24 and 48 h. The expression levels were analyzed using the quantitative RT-PCR method. Expression level in tissues without treatment (0 h) was arbitrarily set to 1 and the levels in tissues from GA3-treated plantlets were given relative to this. Error bars represent standard deviations of mean value from three biological and four technical replicates. ANOVA (analysis of variance) was calculated using SPSS. *P* < 0.05 (*) and *P* < 0.01 (**) was considered statistically significant and extremely significant, respectively

Among the eleven *SmGA2ox* genes, five, including *SmGA2ox2* and *SmGA2ox4*–*SmGA2ox7*, were significantly up-regulated in roots, stems and leaves treated with GA_3_ for 12, 24 and 48 h (Fig. 9). Up-regulation of *GA2ox* genes has been previously found for *AtGA2ox1* and *AtGA2ox2* in *Arabidopsis* treated with exogenous GA_3_ [15] and is considered as a result of feedback control [4, 15]. Additionally, *SmGA2ox1*, *SmGA2ox8*, *SmGA2ox10* and *SmGA2ox11* were significantly down-regulated in all or the majority of the GA_3_-treated tissues analyzed (Fig. 9). *SmGA2ox9* was significantly down-regulated in roots and stems at the time-point of 48 h and significantly up-regulated in roots at the time-point of 12-h-treatment, in stems at the time-points of 12- and 24-h-treatment, and in leaves at the time-points of 24- and 48-h-treatment (Fig. 9v–9x). The expression of *SmGA2ox3* was too low to be detected in roots, stems and leaves (Fig. 8c) and in the tissues of *S. miltiorrhiza* treated with GA_3_. These results suggest that individual members of the *SmGA2ox* gene family exhibit differential responses to GA_3_ treatment and the responses depend on tissue types.

### Responses of *SmCPSs* and *SmKSLs* to exogenous GA_3_

**Fig. 10:**
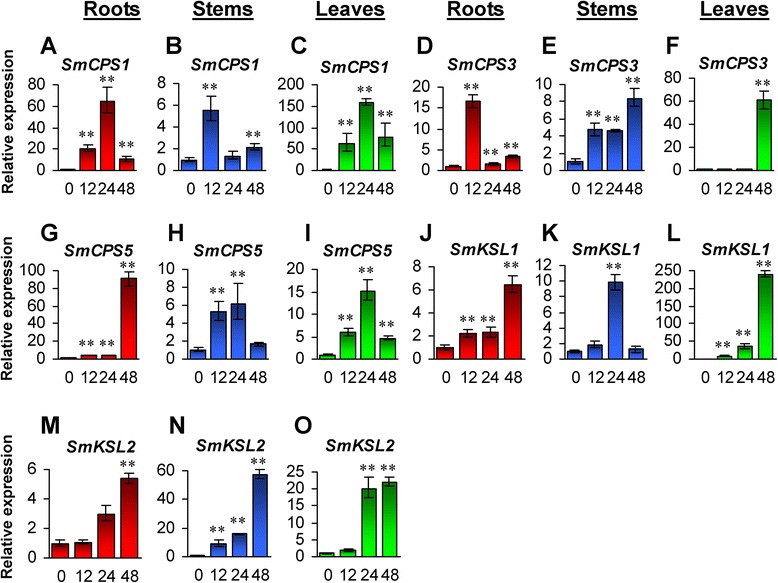
Responses of *SmCPS* and *SmKSL* genes to exogenous GA3 treatment. Fold changes of *SmCPS1* (**a**–**c**), *SmCPS3* (**d**–**f**), *SmCPS5* (**g**–**i**), *SmKSL1* (**j**–**l**) and *SmKSL2* (**m**–**o**) in roots, stems and leaves of *S. miltiorrhiza* plantlets treated with 100 μM GA3 for 0, 12, 24 and 48 h are shown. The expression levels were analyzed using the quantitative RT-PCR method. Expression level in tissues without treatment (0 h) was arbitrarily set to 1 and the levels in tissues from GA3-treated plantlets were given relative to this. Error bars represent standard deviations of mean value from three biological and four technical replicates. ANOVA (analysis of variance) was calculated using SPSS. *P* < 0.05 (*) and *P* < 0.01 (**) was considered statistically significant and extremely significant, respectively

CPS and KS are two enzymes catalyze the formation of *ent*-kaurene from GGPP in two steps via *ent*-copalyl diphosphate (CPP) in the first stage of GA biosynthesis. In our previous studies, five *SmCPS* genes in *S. miltiorrhiza* were identified [12]. Although the function of *SmCPS* genes has not been fully elucidated, *SmCPS1*, which was predominantly expressed in root cortices, is involved in tanshinone biosynthesis [12, 37]. *SmCPS1* were significantly up-regulated in roots, stems and leaves of *S. miltiorrhiza* plants treated with GA_3_ (Fig. 10a–10c). It suggests that tanshinone biosynthesis-related genes may be responsive to exogenous GA_3_ treatment. Similarly, *SmCPS3* and *SmCPS5* were significantly up-regulated in GA_3_-treated plants (Fig. 10d–10i). The expression levels of *SmCPS2* and *SmCPS4* were too low to be detected in the tissues analyzed.

So far, two *SmKSLs*, known as *SmKSL1* and *SmKSL2*, have been identified from *S. miltiorrhiza* [12, 48]. *SmKSL1* showed the highest expression level in stems, followed by leaves, roots and flowers, while the expression levels of *SmKSL2* were similar in all tissues [12, 48]. Previous studies have shown that *SmKSL1* is involved in tanshinone biosynthesis [48]. Analyzing the responses of *SmKSL1* and *SmKSL2* to GA_3_ treatment showed that both *SmKSL1* and *SmKSL2* were significantly up-regulated in roots, stems and leaves of *S. miltiorrhiza* plants treated with exogenous GA_3_ (Fig. 10j–10o), confirming that tanshinone biosynthesis-related genes may be responsive to exogenous GA_3_ treatment.

### Responses of GA metabolism pathway genes to yeast extract and Ag^+^ treatment

**Fig. 11:**
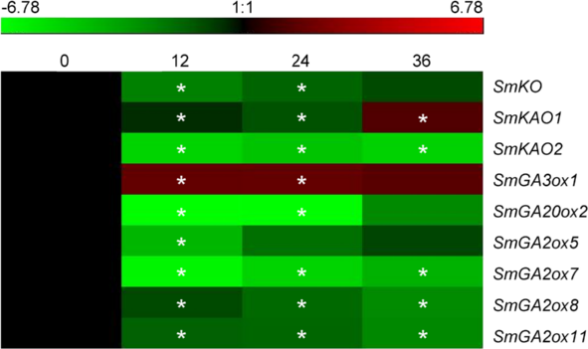
Responses of GA metabolism pathway genes to yeast extract and Ag^*+*^ treatment. *S. miltiorrhiza* hairy roots were treated for 0, 12, 24 and 36 h, respectively. RNA-seq reads were mapped to the cloned ORFs of GA metabolism pathway genes. Genes with RPKM value greater than 1 were analyzed for differential expression using Fisher’s exact test. *P* < 0.05 was considered as differentially expressed. *indicates significant differential expression compared with the level in hairy roots without treatment

In addition to exogenous bioactive GAs, GA metabolism pathway genes may also response to environmental cues, such as light, temperature and various stresses [4]. Yeast extract and Ag^+^ are effective elicitors for the production of terpenoids. Many genes involved in the upstream of GA biosynthesis pathway were significantly up-regulated in *S. miltiorrhiza* after yeast extract and Ag^+^ treatment [48]. However, it was unknown whether the GA metabolism pathway genes were responsive to yeast extract and Ag^+^ treatment. In this study, transcriptome-wide analysis of GA metabolism pathway genes was carried out using RNA-seq data of *S. miltiorrhiza* hairy roots treated with or without yeast extract (100 μg/ml) and Ag^+^ (30 μM) [49]. RNA-seq reads from *S. miltiorrhiza* hairy roots non-treated (0 hpi) and treated for 12 (12 hpi), 24 (24 hpi) and 36 h (36 hpi) were mapped to the cloned ORFs of 22 GA metabolism pathway genes using SOAP2 [50]. A total of nine genes had the RPKM value >1.0 and was considered to be expressed in *S. miltiorrhiza* hairy roots. It includes *SmKO*, *SmKAO1*, *SmKAO2*, *SmGA3ox1*, *SmGA20ox2*, *SmGA2ox5*, *SmGA2ox7*, *SmGA2ox8* and *SmGA2ox11*, of which *SmGA2ox5* was significantly down-regulated at the time-point of 12 hpi, *SmKO* and *SmGA20ox2* were significantly down-regulated at the time-points of 12 and 24 hpi, *SmKAO2*, *SmGA2ox7*, *SmGA2ox8* and *SmGA2ox11* were significantly down-regulated at all time-points of treatment, while *SmGA3ox1* was up-regulated at all time-points of treatment (Fig. 11). *SmKAO1* was down-regulated at the time-points of 12 and 24 hpi and up-regulated at the time-point of 36 hpi (Fig. 11). It suggests that the majority of GA metabolism pathway genes are yeast extract and Ag^+^-responsive.

### Alternative splicing (AS) of GA metabolism pathway genes

**Fig. 12:**
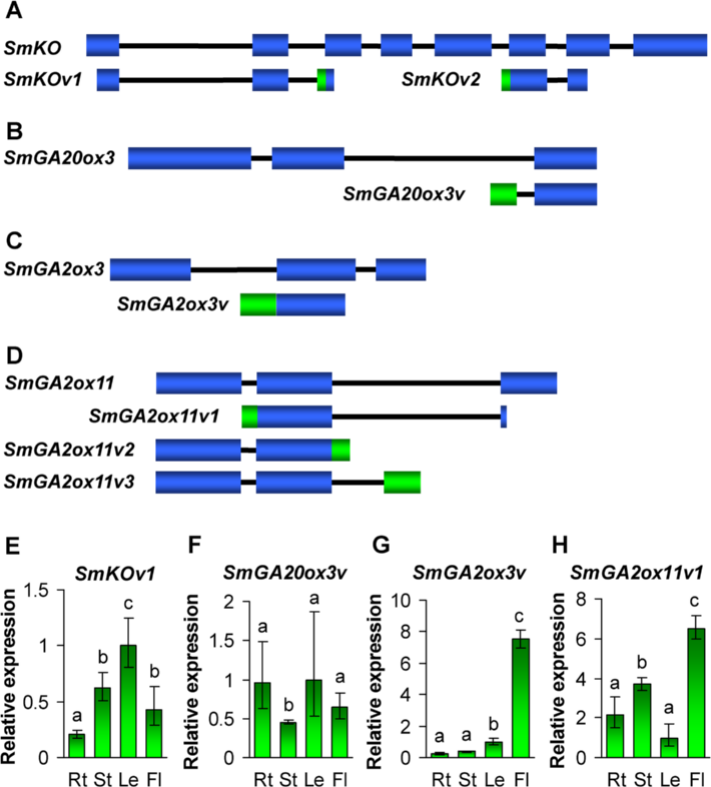
Alternative splicing and tissue-specific expression of various GA metabolism pathway genes *in S. miltiorrhiza*. **a**–**d**: Gene structures of *SmKO* (**a**), *SmGA20ox3* (**b**), *SmGA2ox3* (**c**), *SmGA2ox11* (**d**) and their splice variants. Filled boxes represent exons. Lines represent introns. **e**–**h**: Fold changes of *SmKOv1* (**e**), *SmGA20ox3v* (**f**), *SmGA2ox3v* (**g**) and *SmGA2ox11v1* (**h**) in roots (Rt), stems (St), leaves (Le) and flowers (Fl) of *S. miltiorrhiza* plants. The expression levels were analyzed using the quantitative RT-PCR method. Expression level in leaves was arbitrarily set to 1 and the levels in other tissues were given relative to this. Error bars represent standard deviations of mean value from three biological and four technical replicates. ANOVA (analysis of variance) was calculated using SPSS. *P* < 0.05 was considered statistically significant

Alternative splicing (AS) is a post-transcriptional mechanism of precursor-mRNA (pre-mRNA) [51, 52]. Through AS, multiple distinct mRNA isoforms are produced from a single gene locus. The process of AS plays significant roles in the diversity of transcriptome and proteome and the abundance of transcripts and proteins and is involved in many aspects of plant growth and development, such as photosynthesis, flowering, cereal grain quality, circadian clock, and response to biotic and abiotic stresses [51, 52]. In a previous study, two differently spliced *AtKO* (*GA3*) mRNAs had been cloned, sequenced and detected using Northern hybridization [45]. In order to examine whether genes associated with GA metabolism underwent AS events, we analyzed the assembled *S. miltiorrhiza* transcriptome [42] for the 22 cloned GA metabolism pathway genes. Sequence comparison among unigenes, the cloned cDNAs and the genomic DNA showed that *SmKO*, *SmGA20ox3*, *SmGA2ox3* and *SmGA2ox11* produced splice variants (Fig. 12a–12d). The number of unigenes corresponding to splice variants is two for *SmKO*, one for *SmGA20ox3*, one for *SmGA2ox3*, and three for *SmGA2ox11*, respectively (Fig. 12a–12d).

**Fig. 13:**
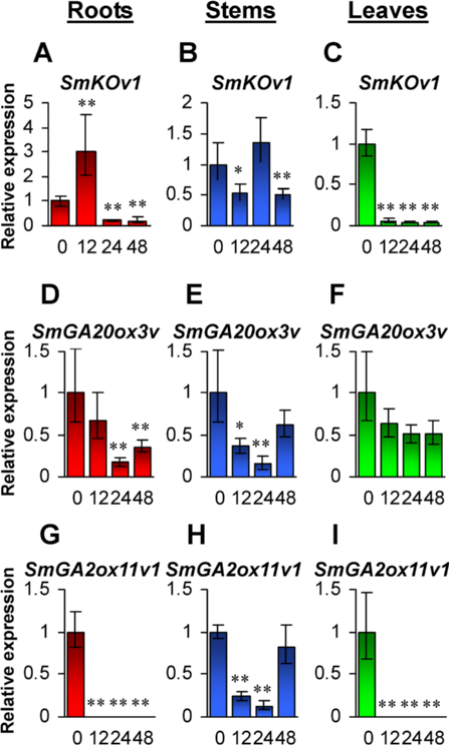
Responses of splice variants to exogenous GA3 treatment. **a**–**i**: Fold changes of *SmKOv1* (**a**–**c**), *SmGA20ox3v* (**d**–**f**) and *SmGA2ox11v1* (**g**–**i**) in roots, stems and leaves of *S. miltiorrhiza* plantlets treated with 100 μM GA3 for 0, 12, 24 and 48 h. The expression levels were analyzed using the quantitative RT-PCR method. Expression level in tissues without treatment (0 h) was arbitrarily set to 1 and the levels in tissues from GA3-treated plantlets were given relative to this. Error bars represent standard deviations of mean value from three biological and four technical replicates. ANOVA (analysis of variance) was calculated using SPSS. *P* < 0.05 (*) and *P* < 0.01 (**) was considered statistically significant and extremely significant, respectively

qRT-PCR analysis using splice variant-specific primers showed that specific amplicons could be obtained for *SmKOv1*, *SmGA20ox3v*, *SmGA2ox3v* and *SmGA2ox11v1* (Fig. 12e–12h). *SmKOv1* exhibited the highest expression in leaves, followed by stems and flowers, and the lowest in roots (Fig. 12e). *SmGA20ox3v* showed higher expression in roots, leaves and flowers than stems (Fig. 12f). *SmGA2ox3v* was predominantly expressed in flowers (Fig. 12g). *SmGA2ox11v1* showed the highest expression in flowers, followed by stems, and the lowest in roots and leaves (Fig. 12h). The expression patterns of splice variants in roots, stems, leaves and flowers were similar to normal transcripts (Figs. 1b, 3d, 8c and 8k), although the overall expression levels of splice variants were significantly lower. *SmKOv1*, *SmGA20ox3v* and *SmGA2ox11v1* showed differential response to exogenous GA_3_ treatment in roots, stems and leaves of *S. miltiorrhiza* (Fig. 13). *SmKOv1* was significantly up-regulated at the time-point of 12-h-treatment, while down-regulated at the time-points of 24- and 48-h-treatment in *S. miltiorrhiza* roots (Fig. 13a). It was significantly down-regulated in stems at the time-points of 12- and 48-h-treatment and in roots at all three time-points of GA_3_ treatment (Fig. 13b and 13c). The responses of *SmKOv1* to GA_3_ treatment were different from *SmKO* (Fig. 1c–1e). *SmGA20ox3v* showed significant down-regulation in roots at the time-points of 24- and 48-h-treatment and in stems at the time-points of 12- and 24-h-treatment, while no significant change was observed in roots at the time-point of 12-h-treatment, in stems at the time-point of 48-h-treatment, and in leaves at all three time-points of GA_3_ treatment (Fig. 13d–13f). *SmGA2ox11v1* exhibited significant down-regulated in roots and leaves at all three time-points and in stems at the time-points of 12- and 24-h-treatment (Fig. 13g–13i). The responsive patterns of *SmGA20ox3v* and *SmGA2ox11v1* were similar to *SmGA20ox3* (Fig. 4g–4i) and *SmGA2ox11* (Fig. 9ab–9ad), respectively. Taken together, these results suggest the importance of AS in regulating GA metabolism in *S. miltiorrhiza*.

## Discussion

### Characterization of the GA metabolism pathway genes in *S. miltiorrhiza*

*S. miltiorrhiza* Bunge is one of the most important Traditional Chinese Medicine (TCM) materials. It is also an emerging model medicinal plant. Identification of GA metabolism pathway genes in *S. miltiorrhiza* may help to elucidate the biological function of these genes, the regulatory mechanism of GA metabolism, and the interplay between the GA metabolism pathway and other terpenoid metabolism pathways. Genome-wide prediction, molecular cloning and expression analysis of the 22 GA metabolism pathway genes encoding SmKO, SmKAO, SmGA20ox, SmGA3ox and SmGA2ox proteins in *S. miltiorrhiza* provide a foundation for further demonstrating the GA metabolism pathway and for commercial application. However, due to the incompleteness of the *S. miltiorrhiza* whole genome assembly, it could be not a complete set of GA metabolism pathway genes.

KO and KAO are two multifunctional cytochrome P450 monooxygenases catalyzing the conversion of *ent*-kaurene to GA_12_ in the second stage of GA metabolism pathway [2, 13, 14]. They are encoded by one or two genes in a plant species. The *Arabidopsis* genome contains an *AtKO* and two *AtKAOs*, while the rice genome has two *OsKOs* and an *OsKAO*. Similarly, from *S. miltiorrhiza*, we identified a *SmKO* and two *SmKAOs*. It is consistent with previous conclusion that enzymes involved in the early steps of the GA metabolism pathway are usually encoded by single or few genes [4]. Analysis of phylogenetic relationship showed that KO and KAO proteins found in a plant species, such as rice OsKO1 and OsKO2, *Coffea arabica* CaKO1 and CaKO2, *Lactuca sativa* LsKO1 and LsKO2, *Arabidopsis* AtKAO1 and AtKAO2, *Pisum sativum* PsKAO1 and PsKAO2 and *S. miltiorrhiza* SmKAO1 and SmKAO2, clustered together in the unrooted neighbor-joining trees (Figs. 1a and 2a). It indicates that KOs and KAOs from a plant species are usually paralogous originating from gene duplication.

Using ChloroP, AtKO had been predicted to contain a plastid transit peptide [17]. Transient expression studies of AtKO-green fluorescent protein (GFP) fusion in tobacco leaves showed that AtKO-GFP was targeted to the chloroplast [17]. In vitro protein import assays with isolated pea chloroplasts showed that AtKO was located on the outer surface of the chloroplast envelope [17]. However, it has been suggested that the subcellular localization of AtKO requires further confirmation, since the strong fluorescence from the chlorophylls may result in false positive results [14]. In this study, subcellular localization analysis of the deduced SmKO, SmKAO1 and SmKAO2 proteins using TargetP 1.1 [53] showed that each of them contained a secretory pathway signal peptide at the N-terminus (Table 1). It indicates that these proteins are most possibly located in the endoplasmic reticulum of *S. miltiorrhiz*a cells. The secretory pathway signal peptides were also found at the N-terminal of KOs and KAOs from *Arabidopsis* and rice (Additional files 2 and 3: Tables S1 and S2). It implies that the conversion of *ent*-kaurene to GA_12_ is also located in the endoplasmic reticulum in *Arabidopsis* and rice. The results are consistent with those from *Arabidopsis* AtKAO1 and AtKAO2 analyses [17] and from early biochemistry studies showing that the conversion of *ent*-kaurenol, *ent*-7α-hydroxyk aurenoic acid and GA_12_ are located in the endoplasmic reticulum [54].

Bioactive gibberellins are converted from GA_12_ through the non-13-hydroxylation pathway and the early 13-hydroxylation pathway under the catalysis of GA20ox and GA3ox [4]. Recently, rice CYP714 members, *CYP714B1* and *CYP714B2*, were reported to encode GA 13-oxidase (GA13ox) catalyzing the conversion of GA_12_ to GA_53_, which is the first step of the early GA 13-hydroxylation pathway [55]. In *Arabidopsis*, a member of the CYP714 member, termed *CYP714A2*, was found to convert GA_12_ to 12α-hydroxyl GA_12_ (GA_**111**_) as a major product and 13-hydroxy GA_12_ (GA_53_) as a minor product, while the other member, termed *CYP714A1*, catalyzed the conversion of GA_12_ to 16-carboxylated GA_12_ [56]. From *S. miltiorrhiza*, a total of nineteen 2-ODDs were identified. It includes six SmGA20oxs and two SmGA3oxs catalyzing the biosynthesis of bioactive GAs and eleven SmGA2oxs involved in the deactivation of bioactive GAs. However, no genomic DNA sequence was retrieved when BLAST analysis of *Arabidopsis* CYP714A1 and CYP714A2 and rice CYP714B1 and CYP714B2 against the current assembly of the *S. miltiorrhiza* genome was carried out using the tBLASTn algorithm [39, 40]. It could be due to the incompleteness of the *S. miltiorrhiza* whole genome assembly or low sequence homology among GA13ox proteins from *S. miltiorrhiza*, *Arabidopsis* and rice.

Among the nineteen *S. miltiorrhiza* 2-ODDs, sixteen were predicted to be located in the cytoplasm. Similarly, 14 of the 16 *Arabidopsis* 2-ODDs and 18 of the 21 rice 2-ODDs were predicted to be located in the cytoplasm. It suggests that the final stage of GA metabolism is conservatively occurred in the cytoplasm. Unexpectedly, various 2-ODDs from *S. miltiorrhiza*, *Arabidopsis* and rice, such as SmGA20ox4, SmGA2ox1, SmGA2ox5, AtGA20ox4, AtGA2ox7, OsGA2ox2, OsGA3ox1 and OsGA2ox11, contain chloroplast transit peptides at the N-terminal. Although the function of these plastid-located 2-ODDs is not clear, some of them may play limited and specific roles in plants. For instance, AtGA20ox5 catalyzes only the first two steps of the successive oxidation reaction sequence [20]. No significant phenotype changes were observed when *AtGA20ox5* was disrupted in *Arabidopsis* plants [20]. Thus, cytoplasm- and plastid-located 2-ODDs probably play distinct physiological roles, which need to be investigated further.

### Multiple layer regulation of GA metabolism in *S. miltiorrhiza*

As one of the five classical phytohormones, GA plays significant roles in many aspects of plant growth and development. Precise regulation of GA content appears to be critical in plant normal development and response to various environmental cues, such as light, temperature and other stresses [4, 33]. Multiple layer regulation may greatly help keeping GA content at the appropriate level and satisfying the needs of cells and organs at different developmental stages and under different environmental conditions. In this study, we showed that GA metabolism in *S. miltiorrhiza* was controlled by multiple mechanisms, such as alternative splicing, tissue-/organ-specific metabolism pathway, positive and negative feedback regulation, and yeast extract and Ag^+^-responsive gene expression.

Alternative splicing represents an important post-transcriptional mechanism in regulation of gene expression during plant growth and development [51, 52]. Transcriptome-wide analysis of splice variants showed the occurrence of AS in more than 40 % of the nine candidate GA metabolism pathway genes with assembled unigenes in *S. miltiorrhiza*. Splice variants showed tissue-specific expression patterns and were responsive to exogenous GA_3_ treatement. These results suggest the importance of AS in regulating GA metabolism in *S. miltiorrhiza*.

Gene expression profiling revealed that the identified GA metabolism pathway genes exhibit tissue-/organ-specific expression patterns. Additionally, exogenous GA_3_ treatment showed that individuals of GA metabolism pathway genes responded differentially and the responses depended on tissue types and organs. Differential expression of GA metabolism pathway genes suggests that each member of a gene family may play different physiological roles. Tissue-/organ-specific expression patterns and response to GA_3_ treatment provide evidence for the hypothesis that an additional layer of regulation may reside in the separation of the GA metabolism pathway into distinct cell types in tissues and organs requiring GA for development [19]. The results indicate the existence of tissue-/organ-specific metabolism pathway involving different enzyme isoforms.

It has been shown that the expression of some GA oxidase genes is under feedback control by bioactive GAs [4, 15, 18, 19, 34–36]. It includes down-regulation of some *GA20ox* and *GA3ox* genes (also known as negative feedback regulation) and up-regulation of some *GA2ox* genes (also known as positive forward regulation). For instance, GA_3_ treatment of *Arabidopsis ga1-2* plants substantially reduced the abundance of *AtGA20ox1*–*AtGA20ox3* transcripts in floral shoots [18]. Consistently, *Arabidopsis AtGA20ox1* (*GA5*) and *AtGA3ox1* (*GA4*) transcription was repressed in wild-type plants treated with exogenous GA_3_ [34, 36] Compared with wild-type plants, expression levels of three potato *StGA20ox* genes, named *StGA20ox1*–*StGA20ox3*, were very much increased in the *ga*_*1*_ dwarf mutant of potato, while treatment of potato *ga*_*1*_ plants with GA_3_ strongly reduced transcript abundance of all three *StGA20ox* genes [35]. In contrast to *AtGA20ox* and *AtGA3ox* genes, expression levels of *Arabidopsis AtGA2ox1* and *AtGA2ox2* were increased by application of GA_3_ [15]. Bioactive GA content reduction caused by *GA20ox* and *GA3ox* gene down-regulation and GA inactivation resulting from *GA2ox* gene up-regulation appear to be important for maintaining active GA concentrations within certain limits [15]. On the other hand, it has been shown that feedback regulation of GA levels does not occur in all organs [14] and the expression of some GA metabolism pathway genes, such as *Arabidopsis AtKO1* [45] and *AtGA3ox2* [57], wheat *TaCPS*, *TaKS*, *TaKO* and *TaKAO* [58], are not subject to feedback regulation by GAs. Down-regulation of *SmGA20ox* and *SmGA3ox* genes and up-regulation of *SmGA2ox* genes suggest the existence of feedback regulation mechanism of GA metabolism in *S. miltiorrhiza*. The underlying mechanism for up-regulation of *SmKO*, *SmKAO1* and *SmKAO2* (positive feedback regulation) and down-regulation of various *SmGA2ox* genes (negative forward regulation) in tissues treated with exogenous GA_3_ remain to be elucidated.

The responses of GA metabolism to environmental cues have been previously analyzed for light and temperature [4, 33]. In this study, we found that nine GA metabolism pathway genes were expressed in hairy roots of *S. miltiorrhiza*. Among them, seven, including *SmKO*, *SmKAO2*, *SmGA20ox2*, *SmGA2ox5*, *SmGA2ox7*, *SmGA2ox8* and *SmGA2ox11*, were significantly down-regulated at different time-points of yeast extract and Ag^+^-treatment. *SmGA2ox1* was significantly up-regulated at all time-points of treatment, while *SmKAO1* was down-regulated at the time-points of 12 and 24 hpi and up-regulated at the time-point of 36 hpi. The identification of yeast extract and Ag^+^-responsive GA metabolism pathway genes provide additional evidence for the regulation of GA metabolism by stresses.

### Crosstalk between GA metabolism and tanshinone biosynthesis in *S. miltiorrhiza*

Both GAs and tanshinones are diterpenoids sharing GGPP as the common precursor [12, 37]. Previously, *SmCPS1* and *SmKSL1* were identified to be key enzyme genes involved in tanshinone biosynthesis in *S. miltiorrhiza* [12, 37, 38]. In this study, both *SmCPS1* and *SmKSL1* were significantly up-regulated in *S. miltiorrhiza* plants treated with GA_3_. Significant change of *SmCPS1* and *SmKSL1* gene expression levels in GA_3_-treated *S. miltiorrhiza* plants suggests tanshinone biosynthesis-related genes were responsive to exogenous GA_3_ treatment. It indicates the existence of crosstalk between GA metabolism and tanshinone biosynthesis. The expression changes of these genes could be important for redirecting GGPP into other branch of the diterpenoid biosynthesis pathway.

## Conclusions

Through systematical identification and characterization, including genome-wide identification, molecular cloning and expression analysis, we showed in this study for the first time a total of 22 candidate GA metabolism pathway genes. It represents five gene families, encoding a SmKO, two SmKAOs, six SmGA20oxs, two SmGA3oxs and eleven SmGA2oxs. Gene feature and phylogenetic analysis showed the conservation and divergence of GA metabolism pathway genes in *S. miltiorrhiza*. Many genes identified in this study were tissue-specifically expressed, feedback-regulated, stress-responsive and alternatively spliced. We found that nine of the twenty two GA metabolism pathway genes were responsive to yeast extract and Ag^+^-treatment in *S. miltiorrhiza* hairy roots and tissue-specifically expressed splice variants existed for *SmKO*, *SmGA20ox3*, *SmGA2ox3* and *SmGA2ox11*. Among the splice variants identified, *SmKOv1*, *SmGA20ox3v* and *SmGA2ox11v1* were GA_3_-responsive. Moreover, we showed that *SmCPS1* and *SmKSL1*, two tanshinone biosynthesis-related genes, were up-regulated in response to GA_3_ treatment. Taken together, our results reveal multiple layer regulation of GA metabolism and crosstalk between GA metabolism and tanshinone biosynthesis in *S. miltiorrhiza*.

## Methods

### Plant materials and GA treatment

*Salvia miltiorrhiza* Bunge (line 993) plants were grown under natural growth conditions in a field nursery at the Institute of Medicinal Plant Development, Chinese Academy of Medical Sciences and Peking Union Medical College, Beijing, China. Roots, stems, leaves and flowers were collected from two-year-old plants and stored immediately in liquid nitrogen until use. Plantlets used for GA treatment were prepared as described previously [12, 42]. Plantlets were sub-cultivated on 1/2 MS agar medium [59] for 6 weeks under a 16/8 h light/dark photoperiod at 25 °C and then transferred to 1/2 MS liquid medium for 2 days. GA_3_ stock solution in 1/2 MS was added to the medium to obtain a final concentration of 100 μM. Plantlets were treated for 12, 24 and 48 h, respectively. Plantlets without GA treatment were used as controls. Leaves, stems and roots from GA-treated plantlets and controls were collected at the same time and stored in liquid nitrogen until use. Three independent biological replicates were performed for each experiment.

### Database search and gene prediction

The deduced amino acid sequences of 19 *Arabidopsis* and 24 rice KO, KAO, GA20ox, GA3ox and GA2ox proteins (Additional files 2 and 3: Tables S1 and S2) were obtained from the *Arabidopsis* Information Resource (TAIR, http://www.arabidopsis.org) and the China Rice Data Center (http://www.ricedata.cn/gene), respectively. *S. miltiorrhiza SmKO*, *SmKAO*, *SmGA20ox*, *SmGA3ox* and *SmGA2ox* genes were predicted as described previously [12, 60, 61]. Briefly, BLAST analysis of *Arabidopsis* and rice KO, KAO, GA20ox, GA3ox and GA2ox amino acid sequences against the *S. miltiorrhiza* genome assembly, which consists of 611,208 contigs representing about 92 % of the entire genome and 96 % of the protein-coding genes [12, 39], were carried out using the tBLASTn algorithm. An e-value cut-off of 1e–5 was applied to the homologue recognition. All retrieved nucleotide sequences encoding proteins with more than 50 % identity to *Arabidopsis* or rice homologs were used for gene prediction on the Genscan web server (http://genes.mit.edu/GENSCAN.html) [41]. The predicted gene models were further examined and corrected manually through comparison with related genes in other plant species identified by BLAST analysis of the retrieved *S. miltiorrhiza* nucleotide sequences against the non-redundant protein sequence (nr) database (http://blast.ncbi.nlm.nih.gov/Blast.cgi) using the BLASTx algorithm with default parameters [40].

### RNA extraction and cDNA cloning

Total RNA was extracted from *S. miltiorrhiza* tissues using the Trizol reagent (Sigma, USA) and the RNA Extract kit (Huayueyang, China). The integrity of total RNA was analyzed on a 1.2 % agarose gel. RNA quantity was determined using a NanoDrop 2000C spectrophotometer (Thermo Scientific, USA). Reverse transcription was performed on total RNA using the SMART RACE cDNA amplification kit (Clontech, USA). cDNA was PCR-amplified under the following conditions: predenaturation at 94 °C for 2 min, 30 cycles of amplification at 94 °C for 30 s, 56 °C for 30 s and 72 °C for 2 min, followed by a final extension at 72 °C for 15 min. Gene specific primers used for full-length cDNA amplifications are listed in Additional file 4: Table S3. Amplicons with expected size were gel-purified, cloned and sequenced.

### Bioinformatic analysis and phylogenetic tree construction

The theoretical isoelectric point (p*I*) and molecular weight (MW) were predicted using the Compute p*I*/MW tool on the ExPASy server (http://web.expasy.org/compute_pi/) [62]. Conserved domains were analyzed by searching the deduced amino acid sequences against the NCBI Conserved Domain Database (CDD, http://www.ncbi.nlm.nih.gov/Structure/cdd/wrpsb.cgi) with the expected E-value threshold of 0.01 and the maximum size of hits to be 1000 amino acids [63]. The localizations of deduced proteins were predicted on the TargetP1.1 server (http://www.cbs.dtu.dk/services/TargetP/) [53]. Phylogenetic trees were constructed for full-length protein sequences using MEGA version 6.0 by the neighbor-joining method with 1,000 bootstrap replicates [64]. The Poisson correction parameter and pairwise deletion of gaps were applied as described previously [12]. For each analysis, only nodes supported by bootstrap values greater than 50 % are shown.

### Quantitative real-time reverse transcription-PCR (qRT-PCR)

DNA contamination removal and reverse transcription was carried out on 1.0 μg total RNA using the RT-PCR kit (Clontech, USA) in a 20 μl volume. The resulting cDNA was diluted to 400 μl with sterile water. PCR was carried out in a 20 μl volume containing 1.0 μl diluted cDNA, 0.8 μl of 10.0 μM forward primer, 0.8 μl of 10.0 μM reverse primer and 10.0 μl 1 × SYBR Premix Ex Taq I (TaKaRa, Japan) using the Bio-Rad CFX96 system (Bio-Rad, USA) under the following conditions: predenaturation at 95 °C for 30 s, 40 cycles of amplification at 95 °C for 5 s, 60 °C for 18 s and 72 °C for 15 s. qPCR was performed in three biological and four technical replicates. Gene-specific primers listed in Additional file 5: Table S4 were designed using Primer-Premier 5.1 (Palo Alto, Canada). The length of amplicons is between 100 and 350 bp. *SmUBQ10* was used as a reference as described previously [12]. The specificity of amplification was assessed by dissociation curve analysis. Results from gene-specific amplification were analyzed using the comparative Cq method which uses an arithmetic formula, 2-ΔΔCq, to achieve results for relative quantification [65]. Cq represents the threshold cycle. Standard derivation was calculated from three independent biological replicates and four technical replicates. Analysis of variance (ANOVA) was calculated using SPSS (Version 21.0, IBM, USA). *P* < 0.05 and *P* < 0.01 were considered as statistically significant and extremely significant, respectively.

### Analysis of yeast extract and Ag^+^-responsive genes

RNA-seq data from *S. miltiorrhiza* hairy roots non-treated (0 hpi) and treated with yeast extract (100 μg/ml) and Ag^+^ (30 μM) for 12 (12 hpi), 24 (24 hpi) and 36 h (36 hpi) were downloaded from GenBank under the accession number SRR924662 [49]. RNA-seq reads were mapped to the cloned ORFs of GA metabolism pathway genes using SOAP2 [50]. The data were analyzed as described previously [42, 61]. Genes with the RPKM (RNA-seq reads mapped to an ORF per total million reads from a treatment per kilobases of the ORF length) value greater than 1 were analyzed for differential expression using Fisher’s exact test as described previously [61]. *P* < 0.05 was considered as differentially expressed.

### Availability of supporting data

*S. miltiorrhiza* GA metabolisms pathway gene sequences supporting the results of this article are available in GenBank under the accession numbers KT853074–KT853095. The other supporting data sets are included within the article and its additional files.

## Additional files

Additional file 1: Figure S1. Conserved domains of enzymes involved in gibberellin metabolism in *S. miltiorrhiza.* Conserved domains of enzymes involved in gibberellin metabolism in *S. miltiorrhiza* are shown. (DOC 183 kb) Additional file 2: Table S1. Sequence features of gibberellin metabolism genes in *A. thaliana.* The accession numbers, cDNA length, number of amino acid residues, molecular weight, p*I* and predicted protein localization are shown. (DOC 59 kb) Additional file 3: Table S2. Sequence features of gibberellin metabolism genes in *O. sativa.* The accession numbers, cDNA length, number of amino acid residues, molecular weight, p*I* and predicted protein localization are shown. (DOC 73 kb) Additional file 4: Table S3. Primers used for full-length coding region cloning. Complete set of primers used for amplication of full-length coding region. (DOC 62 kb) Additional file 5: Table S4. Primers used for qRT-PCR. Complete set of primers used for qRT-PCR. (DOC 87 kb)

## Abbreviations

2-ODD: 2-oxoglutarate-dependent dioxygenase
ANOVA: analysis of variance
AS: alternative splicing
CPP: *ent*-copalyl diphosphate
CPS: *ent*-copalyl diphosphate synthase
GA: gibberellin
GA20ox: GA 20-oxidase
GA2ox: GA 2-oxidase
GA3ox: GA 3-oxidase
GFP: green fluorescent protein
GGPP: *trans*-geranylgeranyl diphosphate
KAO: *ent*-kaurenoic acid oxidase
KO: *ent*-kaurene oxidase
KS: *ent*-kaurene synthase
MEP: 2-C-methyl-D-erythritol 4-phosphate
MVA: mevalonate
MW: molecular weight
ORF: open reading frame
P450: cytochrome P450 monooxygenase
p*I*: isoelectric point
qRT-PCR: quantitative real-time reverse transcription-PCR
TCM: Traditional Chinese Medicine
