# Supplementary material for: Genome-wide analysis, molecular cloning and expression profiling reveal tissue-specifically expressed, feedback-regulated, stress-responsive and alternatively spliced novel genes involved in gibberellin metabolism in Salvia miltiorrhiza
Source: BMC Genomics. 2015 Dec 21;16:1087. doi: 10.1186/s12864-015-2315-5 (PMC4687090; doi:10.1186/s12864-015-2315-5)
Supplement: Additional file 1: Figure S1. — Conserved domains of enzymes involved in gibberellin metabolism in S. miltiorrhiza. Conserved domains of enzymes involved in gibberellin metabolism in S. miltiorrhiza are shown. (DOC 183 kb) [file 12864_2015_2315_MOESM1_ESM.doc]

1 56 513 519

**SmKO**

1 2 432 434

**SmKAO1**

**SmKAO2**

1 477

1 52 153 207 303 346

**SmGA3ox1**


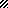

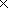


1 46 147 202 299 364


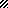

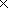


**SmGA3ox2**

**SmGA20ox1**

1 57 159 220 319 378


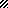

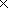


1 63 163 226 325 391


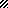

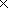


**SmGA20ox2**

1 58 155 226 323 385


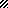

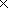


**SmGA20ox3**

1 61 156 223 322 392

**SmGA20ox4**


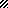

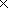


1 45 136 197 296 348


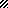

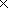


**SmGA20ox5**

1 40 138 196 293 350


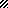

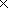


**SmGA20ox6**

1 76 173 230 325 374

**SmGA2ox1**


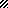

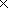


1 25 119 165 270 322


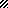

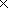


**SmGA2ox2**

1 13 118 165 269 312

**SmGA2ox3**


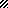

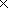


1 37 132 194 289 335


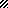

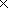


**SmGA2ox4**

**SmGA2ox5**

1 41 136 198 293 334


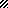

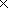


1 34 142 186 281 329


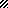

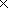


**SmGA2ox6**

1 13 100 162 262 313


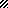

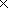


**SmGA2ox7**

1 18 104 168 287 335

**SmGA2ox8**


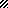

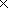


1 21 79 162 261 311


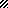

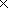


**SmGA2ox9**

1 65 162 214 319 379

**SmGA2ox10**


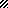

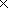


1 25 111 170 272 324


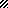

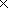


**SmGA2ox11**


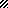

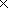


P450 domain

DIOX_N domain

2OG-FeII_Oxy domain

**Figure S1 Conserved domains of enzymes involved in gibberellin metabolism in *S. miltiorrhiza*.** Conserved domains were analyzed by searching the deduced amino acid sequences against the NCBI Conserved Domain Database (CDD, <http://www.ncbi.nlm.nih.gov/Structure/cdd/wrpsb.cgi>). The number of corresponding amino acids is shown.
