# Supplementary material for: Genome-wide analysis, molecular cloning and expression profiling reveal tissue-specifically expressed, feedback-regulated, stress-responsive and alternatively spliced novel genes involved in gibberellin metabolism in Salvia miltiorrhiza
Source: BMC Genomics. 2015 Dec 21;16:1087. doi: 10.1186/s12864-015-2315-5 (PMC4687090; doi:10.1186/s12864-015-2315-5)
Supplement: Additional file 2: Table S1. — Sequence features of gibberellin metabolism genes in A. thaliana. The accession numbers, cDNA length, number of amino acid residues, molecular weight, pI and predicted protein localization are shown. (DOC 59 kb) [file 12864_2015_2315_MOESM2_ESM.doc]

**Table S1 Sequence features of gibberellin metabolism pathway genes in *A. thaliana.***

| **Gene name** | **Accession number** | **cDNA (bp)** | **Len^a^** | **MW^b^ (kDa)** | **p*I*** | **Loc^c^** |
| --- | --- | --- | --- | --- | --- | --- |
| *AtKO* | At5g25900 | 1530 | 509 | 58.16 | 8.27 | S |
| *AtKAO1* | At1g05160 | 1473 | 490 | 56.41 | 8.58 | S |
| *AtKAO2* | At2g32440 | 1470 | 489 | 56.75 | 9.34 | S |
| *AtGA20ox1* | At4g25420 | 1134 | 377 | 43.22 | 5.77 | - |
| *AtGA20ox2* | At5g51810 | 1137 | 378 | 42.94 | 5.61 | - |
| *AtGA20ox3* | At5g07200 | 1143 | 380 | 43.44 | 6.90 | - |
| *AtGA20ox4* | At1g60980 | 1131 | 376 | 43.13 | 7.14 | - |
| *AtGA20ox5* | At1g44090 | 1158 | 385 | 43.16 | 8.04 | C |
| *AtGA2ox1* | At1g78440 | 990 | 329 | 36.73 | 8.53 | - |
| *AtGA2ox2* | At1g30040 | 807 | 268 | 30.15 | 5.60 | - |
| *AtGA2ox3* | At2g34555 | 1008 | 335 | 38.22 | 6.77 | - |
| *AtGA2ox4* | At1g47990 | 966 | 321 | 35.87 | 6.25 | - |
| *AtGA2ox6* | At1g02400 | 990 | 329 | 36.84 | 6.41 | - |
| *AtGA2ox7* | At1g50960 | 1011 | 336 | 38.29 | 6.04 | C |
| *AtGA2ox8* | At4g21200 | 1017 | 338 | 39.09 | 5.88 | - |
| *AtGA3ox1* | At1g15550 | 1077 | 358 | 40.16 | 6.34 | - |
| *AtGA3ox2* | At1g80340 | 1044 | 347 | 38.78 | 6.56 | - |
| *AtGA3ox3* | At4g21690 | 1050 | 349 | 39.21 | 6.16 | - |
| *AtGA3ox4* | At1g80330 | 1068 | 355 | 39.15 | 5.49 | - |

^a^ Len represents the number of amino acid residues.

^b^ MW represents molecular weight.

^c^ Loc represents the protein localization predicted by TargetP1.1. ‘S’ stands for secretory pathway, showing that the sequence cotains a signal peptide. ‘C’ stands for chloroplast, suggesting that the sequence contains a chloroplast transit peptide. ‘-’ indicates any locations other than the plastid, mitochondrion and secretory pathway.
