# Supplementary material for: Genome-wide analysis, molecular cloning and expression profiling reveal tissue-specifically expressed, feedback-regulated, stress-responsive and alternatively spliced novel genes involved in gibberellin metabolism in Salvia miltiorrhiza
Source: BMC Genomics. 2015 Dec 21;16:1087. doi: 10.1186/s12864-015-2315-5 (PMC4687090; doi:10.1186/s12864-015-2315-5)
Supplement: Additional file 4: Table S3. — Primers used for full-length coding region cloning. Complete set of primers used for amplication of full-length coding region. (DOC 62 kb) [file 12864_2015_2315_MOESM4_ESM.doc]

**Table S3 Primers used for full-length coding region cloning.**

| **Gene name** | **Primer name** | **Sequence (5' to 3')** |
| --- | --- | --- |
| *SmKO* | KO-F | CGTGCGGTCTGACGGCGATAGT |
|  | KO -R | CTGCAAACAAAGTTCATCCGATC |
| *SmKAO1* | KAO1-F | CATCACAATTCTTGAAAGGGACT |
|  | KAO1-R | CGTAGTTGCAGAAGATGAT |
| *SmKAO2* | KAO2-F | CTCTCAATACTGTAAGAGAT |
|  | KAO2-R | CTACTTCTAATCTCCAACTAC |
| *SmGA3ox1* | 3OX1-F | GGTTTCACATTTCAAACCCTCC |
|  | 3OX1-R | CAAATGTCACCACAACATC |
| *SmGA3ox2* | 3OX2-F | GAAGAAGCCAAAGCATGCCTTC |
|  | 3OX2-R | GGACGATAGAAGAATTGGT |
| *SmGA20ox1* | 20OX1-F | CTATCATCACACCTCCCAAACTCCT |
|  | 20OX1-R | GTGAAATTACAAAGCTCTCC |
| *SmGA20ox2* | 20OX2-F | CCCATCGGGAACCGCCTATCTG |
|  | 20OX2-R | GCAACAACAATTCAAGGCC |
| *SmGA20ox3* | 20OX3-F | GAACGGAGGATCAGATCAGAGTATC |
|  | 20OX3-R | GAGAGAGAGAGAGATCAACGACAC |
| *SmGA20ox4* | 20OX4-F | GATTCAAGTTATAGGATATGAC |
|  | 20OX4-R | CTCAAGAACATACACAATCTTG |
| *SmGA20ox5* | 20OX5-F | CTAAGTAGAAGGCTCAATGTCCTTCT |
|  | 20OX5-R | CACAATCAACTAATTAAACACGCGCAT |
| *SmGA20ox6* | 20OX6-F | CACTATGATGTCTGCTCACCCTACTC |
|  | 20OX6-R | CCCTGGCTATATAGGTAGGTAGGT |
| *SmGA2ox1* | 2OX1-F | CTAATACGACTCACTATAGGGCA |
|  | 2OX1-R | GATGATAAATATGTGGAGGCCACAT |
| *SmGA2ox2* | 2OX2-F | GATCCTATAAATGCAAATAATCTAGC |
|  | 2OX2-R | CATGCAACACCAAACTGTAGTACAG |
| *SmGA2ox3* | 2OX3-F | CTTGTTGCAAGTATATCCATAGAGCGAG |
|  | 2OX3-R | GTAGAACATAAGCATGAAGCAACAAGT |
| *SmGA2ox4* | 2OX4-F | CATAAACAAACTGCCACACGTG |
|  | 2OX4-R | GGGGATCTAGTCTTATTCCCTT |
| *SmGa2ox5* | 2OX5-F | GAATGAGCTCTTCACACAATTTC |
|  | 2OX5-R | GCACGAACACGAACACGAACAC |
| *SmGA2ox6* | 2OX6-F | GTACACAAAATTAATCATGGTA |
|  | 2OX6-R | CCTACTACCAAAAACTCG |
| *SmGA2ox7* | 2OX7-F | CACCAACACTCTTCTCTCTCTAC |
|  | 2OX7-R | GATGAAAACTTAAGGAGTAGGTGAC |
| *SmGA2ox8* | 2OX8-F | ATCGTTGAAGGTGGTCGCC |
|  | 2OX8-R | GAACATGTACATGTGCAACTACACCC |
| *SmGA2ox9* | 2OX9-F | CCTCGTCTCTCCCCACTTCCTCTAT |
|  | 2OX9-R | GTGATGAGAGGGCAATGCAAAGAGAT |
| *SmGA2ox10* | 2OX10-F | GCCAGATGTCTCTCTATAGTTTCT |
|  | 2OX10-R | GAGAGAGAGAGAGAGGAAATCTAT |
| *SmGA2ox11* | 2OX11-F | GATACATTGTACCACTTCACAC |
|  | 2OX11-R | CTATTATCGGCCAATTTTGAC |
