# Supplementary material for: Genome-wide analysis, molecular cloning and expression profiling reveal tissue-specifically expressed, feedback-regulated, stress-responsive and alternatively spliced novel genes involved in gibberellin metabolism in Salvia miltiorrhiza
Source: BMC Genomics. 2015 Dec 21;16:1087. doi: 10.1186/s12864-015-2315-5 (PMC4687090; doi:10.1186/s12864-015-2315-5)
Supplement: Additional file 5: Table S4. — Primers used for qRT-PCR. Complete set of primers used for qRT-PCR. (DOC 87 kb) [file 12864_2015_2315_MOESM5_ESM.doc]

**Table S4 Primers used for qRT-PCR.**

| **Gene name** | **Primer name** | **Sequence (5' to 3')** |
| --- | --- | --- |
| *SmUBQ_10_* | UBQ_10_-RT-F | agatgggcggacacttgctgatta |
|  | UBQ_10_-RT-R | actctccacctccaaagtgatggt |
| *SmCPS1* | CPS1-RT-F | ccacatcgccttcagggaagaaat |
|  | CPS1-RT-R | tttatgctcgatttcgctgcgatct |
| *SmCPS2* | CPS2-RT-F | ggtctcatcgccttcaacgaagat |
|  | CPS2-RT-R | tccttatcctttatgctcccatcca |
| *SmCPS3* | CPS3-RT-F | ggagatgccaattcgaacatcaga |
|  | CPS3-RT-R | tcaaatatagttgcggcggccaaa |
| *SmCPS4* | CPS4-RT-F | cggctgccttgggctacaacaata |
|  | CPS4-RT-R | tccctggtgacctcctccttccca |
| *SmCPS5* | CPS5-RT-F | tagaagatgcagctactttctctgct |
|  | CPS5-RT-R | catcatcttcaccgccgtactgtt |
| *SmKSL1* | KSL1-RT-F | tggaaacagtgtgacccttctgct |
|  | KSL1-RT-R | gcttgcatacaaataacacccaatcct |
| *SmKSL2* | KSL2-RT-F | ttagttttggagggcaagaagagtgt |
|  | KSL2-RT-R | ctcctgtttggtcgttgagaagaata |
| *SmKO* | KO-RT-F | GCTTCTGGCAAGGCAATCAACTGT |
|  | KO-RT-R | CCTCGTTGAGTTGGTCGAAT |
| *SmKAO1* | KAO1-RT-F | GCGTTCGAGCAATGGCAATCAACAT |
|  | KAO1-RT-R | CCCTTGTCATCTTGAGCATCCATC |
| *SmKAO2* | KAO2-RT-F | GCTGCAGATGAGAAGGGTAGAAG |
|  | KAO2-RT-R | CCAGCTGGTCTGTTTCTCACAGTT |
| *SmGA3ox1* | 3OX1-RT-F | CCGATTCGTTACTACTGACGATC |
|  | 3OX1-RT-R | GGACGACACGATGGCACATAGT |
| *SmGA3ox2* | 3OX2-RT-F | GACTCAACCATCCTCACGATCCT |
|  | 3OX2-RT-R | CAGGTACGCCACCGAGAAAC |
| *SmGA20ox1* | 20OX1-RT-F | GATCCGACATCACTGACCATCCT |
|  | 20OX1-RT-R | GGATTATCGTCGTCCACCAACTCAT |
| *SmGA20ox2* | 20OX2-RT-F | GATGAGCAGACTGTCGCTAGGGAT |
|  | 20OX2-RT-R | GCAGCTCTTGTACCTGGCGTTGGAT |
| *SmGA20ox3* | 20OX3-RT-F | GAACAAGGAGAGGGTTCGGAGAT |
|  | 20OX3-RT-R | GGAAGCGAATAATGCTGTGTCT |
| *SmGA20ox4* | 20OX4-RT-F | GGTGACACATTCATGGCAGTAT |
|  | 20OX4-RT-R | GGTATTCATGTCTGCCCTGTAGT |
| *SmGA20ox5* | 20OX5-RT-F | CACCTTCATGGCGCTGTCGAAT |
|  | 20OX5-RT-R | GGTAGTGCTTCTGCGTGAACT |
| *SmGA20ox6* | 20OX6-RT-F | CTCCGCATTATCGAATGGAGTGT |
|  | 20OX6-RT-R | GGAGTGTTGCAACATCAACCCTAT |
| *SmGA2ox1* | 2OX1-RT-F | CTGTTTCAGGCATGGAGCAACAACCT |
|  | 2OX1-RT-R | CCAATGCTCTGAACATCCACCTCGATCT |
| *SmGA2ox2* | 2OX2-RT-F | GATTGTCTGCAGGTGATGAGCAAT |
|  | 2OX2-RT-R | GAGACCAAGTGAATTCGTGGTAC |
| *SmGA2ox3* | 2OX3-RT-F | GTGACGTCTTGCAGGCTATGACGAAT |
|  | 2OX3-RT-R | CCAACTGAAGCTTCGATACGTAGGAT |
| *SmGA2ox4* | 2OX4-RT-F | GAGTGTGGAGCATAAAGTGATGGCT |
|  | 2OX4-RT-R | GAATCTTCTTGGACTTTGGCTCTG |
| *SmGA2ox5* | 2OX5-RT-F | CATGATGCAGGCCATGAGCGACGAT |
|  | 2OX5-RT-R | GAGGGTCTTGAGGTCGAGTTCATTCT |
| *SmGA2ox6* | 2OX6-RT-F | GGGACTTGTTTCAGCGTGGAGTAAT |
|  | 2OX6-RT-R | GTAGGAGAAACCTTGGGAGTCCAA |
| *SmGA2ox7* | 2OX7-RT-F | GTGACTCCTTGCAGGTGATGACT |
|  | 2OX7-RT-R | CAGCCAGCCTTGAATTGTAAACAG |
| *SmGA2ox8* | 2OX8-RT-F | CACCAACGGCAGATTCACGAGCGT |
|  | 2OX8-RT-R | GAGGTCGAGACGATGATCTGCCAAT |
| *SmGA2ox9* | 2OX9-RT-F | CGACATCTTACGGGTAATGACGAATGG |
|  | 2OX9-RT-R | CAGCTTGCTTGTATTCACCCCAAGTG |
| *SmGA2ox10* | 2OX10-RT-F | GGATTCGCTGCAGGTGATGACTAAC |
|  | 2OX10-RT-R | CCTTGTCTTGTAGGCTGACTTCTTGT |
| *SmGA2ox11* | 2OX11-RT-F | GCGACTCCTTACAAGTGATGACT |
|  | 2OX11-RT-R | CCCCTTGCATGAGTGATGCCAAT |
| *SmKOv1* | KOv1-RT-F | GCCTAAGGTACCAGGCTTACCAGT |
|  | KOv1-RT-R | CCTAAAGCAAGACAAATCATGTATG |
| *SmGA20ox3v* | 20ox3v-RT-F | CCACTTAGGCTAACTGGCTAAGTG |
|  | 20ox3v-RT-R | CTCACCACCTTGTCCTCATTAG |
| *SmGA2ox3v* | 2ox3v-RT-F | GGTGGTACGTGCCTGATTTGTC |
|  | 2ox3v-RT-R | CTACGATCAGATCCAATATCTCAC |
| *SmGA2ox11v1* | 2ox11v1-RT-F | CGTTGCGTTCGGCTTCTGACTTG |
|  | 2ox11v1-RT-R | GAGATGATCTGAGGGTCGGTGTG |
